# Robust Benchmark Structural Variant Calls of An Asian Using State-of-the-art Long-read Sequencing Technologies

**DOI:** 10.1016/j.gpb.2020.10.006

**Published:** 2021-03-02

**Authors:** Xiao Du, Lili Li, Fan Liang, Sanyang Liu, Wenxin Zhang, Shuai Sun, Yuhui Sun, Fei Fan, Linying Wang, Xinming Liang, Weijin Qiu, Guangyi Fan, Ou Wang, Weifei Yang, Jiezhong Zhang, Yuhui Xiao, Yang Wang, Depeng Wang, Shoufang Qu, Fang Chen, Jie Huang

**Affiliations:** 1 National Institutes for food and drug Control (NIFDC), Beijing 10050, China; 2 BGI-Qingdao, BGI-Shenzhen, Qingdao 266555, China; 3 GrandOmics Biosciences, Beijing 102200, China; 4 Annoroad Gene Technology (Beijing) Co., Ltd., Beijing 102200, China; 5 BGI-Shenzhen, Shenzhen 518083, China; 6 MGI, BGI-Shenzhen, Shenzhen 518083, China; 7 State Key Laboratory of Agricultural Genomics, BGI-Shenzhen, Shenzhen 518083, China; 8 China National GeneBank, BGI-Shenzhen, Shenzhen 518120, China

**Keywords:** Asian benchmark, Reference material, Structural variation, Haplotype-resolved, Sanger validation

## Abstract

The importance of structural variants (SVs) for human phenotypes and diseases is now recognized. Although a variety of SV detection platforms and strategies that vary in sensitivity and specificity have been developed, few benchmarking procedures are available to confidently assess their performances in biological and clinical research. To facilitate the validation and application of these SV detection approaches, we established an Asian **reference material** by characterizing the genome of an Epstein-Barr virus (EBV)-immortalized B lymphocyte line along with identified benchmark regions and high-confidence SV calls. We established a high-confidence SV callset with 8938 SVs by integrating four alignment-based SV callers, including 109× Pacific Biosciences (PacBio) continuous long reads (CLRs), 22× PacBio circular consensus sequencing (CCS) reads, 104× Oxford Nanopore Technologies (ONT) long reads, and 114× Bionano optical mapping platform, and one *de novo* assembly-based SV caller using CCS reads. A total of 544 randomly selected SVs were validated by PCR amplification and Sanger sequencing, demonstrating the robustness of our SV calls. Combining trio-binning-based haplotype assemblies, we established an SV benchmark for identifying false negatives and false positives by constructing the continuous high-confidence regions (CHCRs), which covered 1.46 gigabase pairs (Gb) and 6882 SVs supported by at least one diploid haplotype assembly. Establishing high-confidence SV calls for a benchmark sample that has been characterized by multiple technologies provides a valuable resource for investigating SVs in human biology, disease, and clinical research.

## Introduction

Structural variants (SVs) are generally defined as genomic changes spanning at least 50 bp, including deletions, insertions, duplications, inversions, and translocations [1]. They contribute to the diversity and evolution of human genomes at individual and population levels [2], [3]. Owing to their large size, SVs often exert greater impacts on gene functions and phenotypic changes than small variants [4], [5], [6], [7]. The importance of SVs has been highlighted by their contribution to human diseases including cardiovascular diseases [8], autism [9], and a range of other disorders [10]. Therefore, it is crucial to systematically profile SVs in the human genome for both biological and clinical studies.

There are no gold-standard benchmarking procedures for SVs from next-generation sequencing (NGS) platforms. SVs from NGS platforms are largely inferred from indirect evidence of disturbance of read mapping around the variation. Since SVs tend to reside within repetitive DNA and often span more base pairs than short reads (< 1000 bp), the short reads of NGS usually lack sensitivity, leading to inevitable challenges in SV detection [11], [12]. Moreover, SV detection approaches vary in both sensitivity and specificity, as they emphasize different SV-dependent and library-dependent features. Accurate identification of structural variation is very complex; it requires the characterization of the multifaceted features of SVs, including sequence information, type of variation, length, and location of breakpoints. As a result, different SV callers make inconsistent predictions [12], [13]. Therefore, owing to the complexity of SVs and the inconsistency of different SV callers, a comprehensive assessment of SV detection has been problematic.

Several efforts have been made to benchmark SV calls. The Genome in a Bottle Consortium (GIAB), hosted by the National Institute of Standards and Technology (NIST), started building high-quality benchmark SV calls in 2016. They distributed a set of 2676 high-confidence deletions and 68 high-confidence insertions using SVClassify for the pilot genome NA12878 [14], which had been released as NIST reference material 8398. Recently, GIAB released a more comprehensive SV benchmark set for the Ashkenazi Jewish son NA24385 (NIST RM8391) with 2.66 gigabase pairs (Gb) of benchmark regions and 9641 high-confidence SVs supported by at least one diploid assembly; however, the identified SVs were not validated by experimental methods such as Sanger sequencing [15]. A well-characterized SV benchmark is valuable in identifying false positive and false negative SVs called by various platforms and approaches. Yet, so far we don’t have an Asian-specific SV benchmark. The gnomAD-SV, comprising SVs from 14,891 genomes, reveals that different continental populations exhibit different levels of genetic diversity and SV features [16]. Therefore, designing an Asian benchmark is very necessary for promoting Asian genomic and disease research.

Our work is aimed at designing an Asian reference material comprising identified benchmark regions and high-confidence SV calls. This Asian benchmark would be valuable for Asian studies in three aspects. First, it provides physical material basis for Asian genomic and clinical research by collecting and preserving Asian genetic resources, accessible for Asian-specific biological testing and drug screening. Second, the benchmark SV calls for a characterized cell line will serve as a gold standard for evaluating the performance of diverse SV detection platforms or strategies, including NGS and long-read sequencing technologies. Third, this set of standards will become a threshold for clinical testing and help validate SV detection approaches in clinical practice. Based on the design of this benchmark, future benchmarks comprising pathogenic SVs could be developed for the clinical diagnosis of SV-related diseases.

Establishment of immortalized cell lines is a routine strategy for building a reference material for biological research and clinical practice. The immortalized B lymphocyte line transformed by Epstein-Barr virus (EBV) is a mainstream approach used by international genetic storage institutions, including the NIGMS Human Genetic Cell Repository and the UK Biobank. EBV infection leads to B lymphocyte proliferation and immortalization *in vitro*, resulting in the establishment of immortalized B lymphocyte lines. The immortalized B lymphocytes potentially provide unlimited genomic DNA resources and have been extensively used as a biological source for genetic and medical studies [17]. Previous studies suggest that EBV exists in the episomal form and is not integrated into the host cell chromosome, maintaining the host genome intact [18], [19], [20].

**Figure 1:**
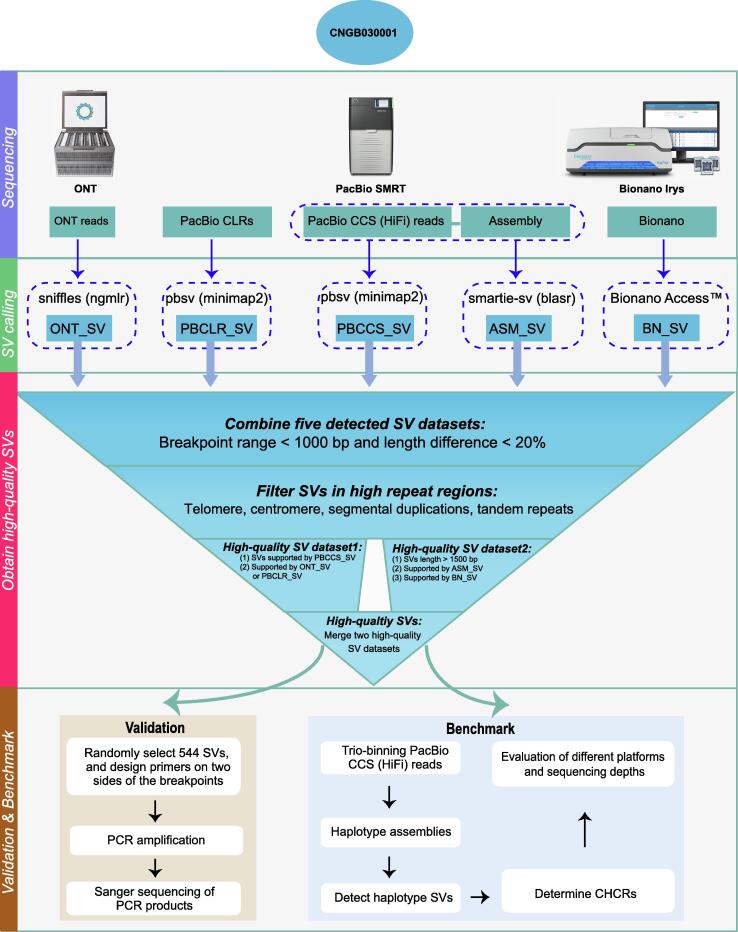
**Workflow for establishing the SV benchmark by integrating different long-read sequencing technologies and approaches** The established reference material CNGB030001 was sequenced by ONT (ONT reads), PacBio SMRT (PacBio CLRs and PacBio CCS reads), and Bionano Irys platforms. SVs were called using four corresponding alignment-based approaches and one CCS assembly-based approach. See Materials and methods for details of the callers and settings. A high-confidence SV callset was constructed by filtering and integrating five candidate SV callsets according to the criteria illustrated in the inverted triangle. A subset comprising 544 randomly-selected SVs from the high-confidence SV callset was validated by PCR amplification and Sanger sequencing. A final SV benchmark located on CHCRs was established by retaining the SVs supported by diploid assemblies. SV, structural variant; ONT, Oxford Nanopore Technologies; PacBio, Pacific BioSciences; SMRT, Single Molecule Real Time; CLR, continuous long read; CCS, circular consensus sequencing; ASM_SV, assembly-based SV calls; BN_SV, Bionano-based SV calls; CHCR, continuous high-confidence region.

The advent of long-read sequencing technologies has greatly aided SV characterization. Although different long-read sequencing platforms apply diverse technologies, they are different from NGS by producing very long reads (1–100 kb). In contrast to the NGS short reads, the long reads provide an advantageous potential to increase the reliability and resolution of SV detection [21]. Given the advantages of long reads, our work established a high-confidence Asian SV benchmark for deletions and insertions by establishing an EBV-immortalized B lymphocyte line and characterizing its genome. We performed large-scale SV benchmarking across a range of the latest long-read sequencing or optical mapping techniques, including Pacific Biosciences (PacBio) continuous long reads (CLRs), PacBio circular consensus sequencing (CCS) reads, Oxford Nanopore Technologies (ONT) long reads, and Bionano optical mapping (Figure 1). After comparing the performances of different platforms, we integrated and genotyped the final SV callset. Sanger sequencing validated the high confidence of our SV calls. We assembled haplotype-resolved diploid genomes via a trio-binning approach using the PacBio CCS reads, and only high-confidence SVs supported by at least one diploid haplotype assembly were retained in the SV benchmark. The established cell line and SV benchmark will provide a standard for assessing the precision and accuracy of different SV detection approaches, and ensure delivering accurate and reliable results for biological and genomic research on Asians. The immortalized B lymphocyte line will serve as an unlimited resource of Asian genomic DNA that can be extensively used in future SV and medical studies.

## Results

### Establishment of an immortalized B lymphocyte line

The peripheral venous blood B lymphocytes of a healthy Chinese man from Beijing, China were collected and infected with EBV, which led to B lymphocyte proliferation and subsequent immortalization *in vitro*. Lymphocytes were treated with cyclosporine A to increase the immortalization efficiency [17]. The morphology of the transformed cells was checked. Then, the transformed cells were passaged continuously under a sterile environment and frozen for storage. Cells grew well after resuscitation, and resuscitation experiments showed typical cell deformation and clonal growth characteristics. Finally, an immortalized B lymphocyte line (CNGB030001) was successfully established.

### Sequencing by long-read sequencing platforms

By sequencing the immortalized cells, we generated 312.77 Gb (∼ 104×) ONT data, 326.98 Gb (∼ 109×) PacBio CLR data, and 341.67 Gb (∼ 114×) Bionano data (Table 1). Compared to the PacBio CLR, ONT displayed a similar sequencing accuracy rate but an obviously longer read length (CLR: 9.2 kb *vs*. ONT: 24.6 kb). In addition, we obtained ∼ 22× highly accurate PacBio CCS (HiFi) reads, after error correction from 869.48 Gb raw data (∼ 266×). The percentage of Q20 (accuracy rate: 99%) of the total CCS reads was 67.6% with an average read length of 12.0 kb, providing a high-quality foundation for SV calling. According to the read length–GC plots, these four platforms performed very well in terms of uniformity of read length and GC content (Figures S1–S4).

**Table 1:** **Summary of sequencing results for different platforms**

**Platform**	**Cell number**	**Sequencing type**	**Total base number (Gb)**	**Depth**	**Read length (average ± SD, bp)**	**Read accuracy**	**GC content (average ± SD, %)**
PacBio CLR	31	Subreads	326.98	109**×**	9212 ± 6984	0.8	42.66 ± 5.82
PacBio CCS	24	CCS	869.48; 71.85	266**×**; 22**×**	11,961 ± 3662	0.987 ± 0.019	40.59 ± 5.63
ONT	8	1D	312.77	104**×**	24,589 ± 22,597	0.849 ± 0.028	40.79 ± 5.55
Bionano	1	BspQ1	341.67	114**×**	-	-	-

*Note*: For PacBio CCS reads, 869.48 Gb and 266× refer to the total base number and the depth of the raw data, respectively, while 71.85 Gb and 22× refer to the total base number and the depth of CCS data after the error correction. For PacBio CLR subreads, base quality of the newer PacBio Sequel Sequencer was not available, so we used an empirical read quality of 0.8. PacBio, Pacific BioSciences; CLR, continuous long read; CCS, circular consensus sequencing; ONT, Oxford Nanopore Technologies; Gb, gigabase pair.

### Candidate insertions and deletions called from different platforms

High accuracy is the prerequisite for establishing SV benchmark. For accuracy, we focused on detecting and characterizing large insertions and deletions (Figure 1). By aligning PacBio CLR subreads, we identified 5871 deletions and 6936 insertions (Table 2). The size distribution of deletions displayed 300 bp and 6 kb peaks related to SINE-Alu and LINE elements, respectively (Figure S5), implying effective SV calling by long reads [22], [23]. By aligning PacBio CCS reads, we identified 17,901 SVs including 8317 deletions and 9584 insertions (Table 2; Figure S6). Compared to PacBio CLR, most of the additional SVs from PacBio CCS were 50–100 bp deletions. Similar to the PacBio CLR result, both SINE-Alu and LINE deletions were identified; however, no LINE elements for insertions were found in the CCS SV calls, probably due to the limitation of PacBio read length (Figure S6).

**Table 2:** **Insertions and deletions identified by different calling approaches**

**Data type**	**Calling method**	**Deletion**				**Insertion**			
		**Count**	**Minimum length (bp)**	**Maximum length (bp)**	**Average length (bp)**	**Count**	**Minimum length (bp)**	**Maximum length (bp)**	**Average length (bp)**
PacBio CLR	minimap2 + pbsv	5871	50	45,516	562	6936	50	10,273	403
PacBio CCS	minimap2 + pbsv	8317	50	75,746	480	9584	50	9805	470
ONT	ngmlr + sniffles	7668	50	62,462,724	90,997	6717	50	7750	326
Bionano	Electronic mapping	1517	224	4,407,162	65,053	3241	231	970,567	6404
CCS assembly	blasr + smartie-sv	10,345	50	75,030	788	17,382	50	64,449	611

The average read length of ONT data is longer than that of PacBio, and the Bionano optical mapping relies on the density of restriction sites on the genome [24]; therefore, theoretically they can efficiently detect the 6 kb LINE elements for insertions. We detected 14,385 SVs (including 7668 deletions and 6717 insertions) by ONT, as well as 4758 SVs (including 1517 deletions and 3241 insertions) by Bionano (Table 2). Both ONT and Bionano successfully observed a LINE insertion peak of ∼ 6 kb (Figures S7 and S8), but Bionano failed to detect the two short SINE-Alu events for deletions and insertions.

Apart from the alignment strategies, a *de novo* assembly-based method was also applied for SV calling. We performed a *de novo* assembly using 22× PacBio CCS reads, producing 3542 contigs with the maximum length of 72 Mb and the N50 of 13 Mb. Good collinearity was observed from aligning the assembled contigs against the reference genome, indicating that no visible structural errors were introduced in the assembly (Figure S9). Finally, we detected 27,727 SVs using smartie-sv [25], which were more than those from alignment-based approaches (Table 2). The increase was mainly from small-scale insertions and deletions. Most noteworthy, the expected four insertion and deletion peaks related to SINE-Alu and LINE elements were all observed in the assembly-based SV calls (Figure S10).

### Unique and common SVs among different platforms

**Figure 2:**
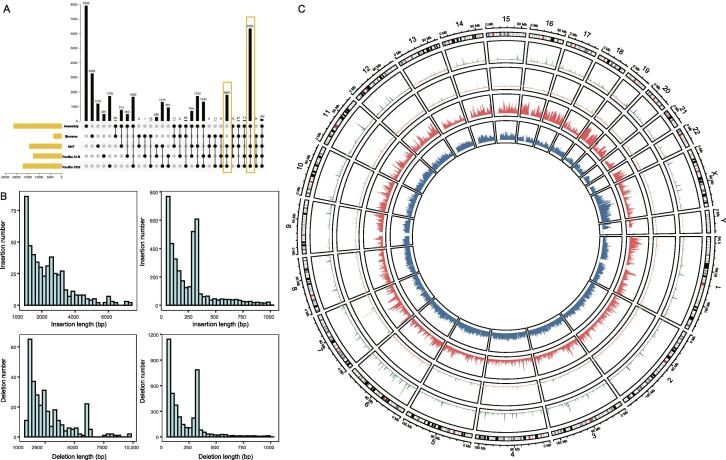
**Comparison of candidate SV callsets from different technologies and characterization of the high-confidence SV callset** **A.** Counts of common SVs among five candidate SV callsets from multiple approaches. **B.** Size distributions in 0–1000 bp and 1000–8000 bp ranges for insertions and deletions in the high-confidence SV callset. Distributions display 300 bp and 6 kb peaks related to SINE-Alu and LINE elements, respectively. **C.** Circos plot illustrating the distributions of deletions, insertions, and repeat elements of the high-confidence SV callset using sliding non-overlapping windows of 1 Mb across all chromosomes of the human genome. From the outer circle to the inner circle, the four circles represent the counts of deletions, insertions, SINE/Alu, and LINE per 1 Mb window, respectively.

None of the approaches was comprehensive in SV discovery, as a significant fraction of the identified SVs was unique to a particular approach. The counts of unique SVs and common SVs among different SV calling approaches are summarized in Figure 2A. PacBio CLR possessed the least unique SVs (491), and the CCS assembly-based approach had the most unique SVs (7930). Due to the specificity of Bionano which was not accurate at the base resolution, there were only 160 SVs shared by all five calling approaches. With Bionano excluded, the three alignment-based single-molecular sequencing approaches (PacBio CLR, PacBio CCS, and ONT) showed high consistency of SV calls with 8156 common SVs. After integrating the CCS assembly-based result, the total number of common SVs reached 6355 for the four datasets.

### A high-confidence SV callset constructed by integrating platforms

We integrated the aforementioned candidate SV calls to construct a high-confidence SV callset by following specific steps and criteria (Figure 1). Considering the features of different sequencing platforms, there were two main reasons for applying these criteria. First, the outstanding long-read sequencing capacities of ONT, PacBio CLR, and Bionano guaranteed the longest possible read length, facilitating successful cover of large SVs. Second, PacBio CCS and CCS assembly-based approach emphasized the high accuracy of SVs. The longest possible read length and high accuracy guaranteed the high confidence of final SV calls. After filtering and integrating, a callset comprising 8938 high-confidence SVs was established. SV distributions across autosome chromosomes showed that the number of the distributed SVs had a good linear correlation with the chromosome length (*R*^2^ = 0.85, *P* < 0.0001, Figure S11).

We examined the support for high-confidence SV calls from different sequencing platforms. Bionano showed the lowest support with only 250 common SVs with the high-confidence SV calls, and the alignment-based PacBio CCS displayed the highest support with 8914 common SVs. The CCS assembly-based approach (6419), PacBio CLR (6797), and ONT (7603) showed similarly high support (Figure S12). The length distributions of high-confidence SVs clearly revealed four SINE-Alu and LINE peaks (Figure 2B). Moreover, the distribution of insertions and deletions in each chromosome was consistent with the density of SINE-Alu and LINE elements (Figure 2C; Table S1).

### PCR and Sanger sequencing validated the high-confidence SV callset

**Figure 3:**
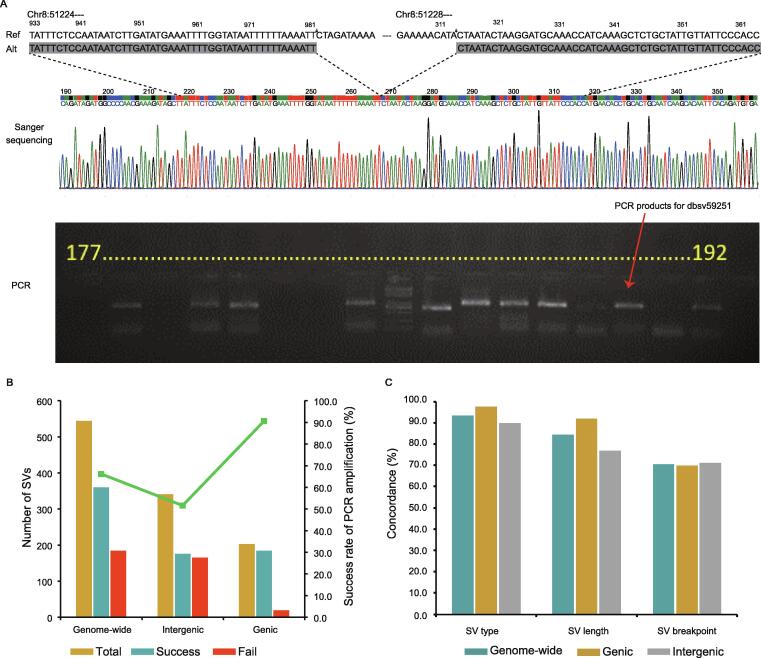
**PCR amplification and Sanger sequencing validated the high-confidence SVs** **A.** An example of Sanger sequencing validating a deletion event in chromosome 8. **B.** PCR amplification rates for different genomic regions. The green line represents the amplification success rate. **C.** Consistency rates of SV type, SV length, and breakpoint position between the Sanger sequenced SVs and the high-confidence SVs. Uncertain sites were excluded.

To validate the accuracy of the high-confidence SV calls, 400 SVs were randomly selected from the 8938 SVs for performing PCR amplification and paired-end Sanger sequencing (Figure 3A). Of the 400 SVs, 244 were successfully amplified. We next randomly selected a second batch of 200 SVs that contained 56 amplification-failed SVs from the first batch and 144 new SVs. This time 22 of the 56 amplification-failed SVs were successfully amplified after PCR primer re-design. Of the 544 SVs assessed by PCR, 203 and 341 were located in the genic and intergenic regions, respectively. In total, 360 SVs were successfully amplified, and the overall amplification success rate was 66.2%, with the amplification success rate within genes reaching 90.6% — notably higher than 51.6% in intergenic regions (Figure 3B). This result is not unexpected, as PCR amplification tends to be hindered by complex regions, such as repetitive abundant regions. Moreover, we analyzed the length of amplified SVs and found that smaller-size SVs had higher amplification rates than larger-size SVs (Figure S13).

Among 360 amplification sites, 317 (∼ 88.1%) were successfully sequenced by paired-end Sanger sequencing and aligned to the reference genome (Figure 3A). The sequenced SVs were compared to the high-confidence SVs to check the SV type, SV length, and breakpoint position separately (Figure S14). The consistency of length or breakpoint position was assessed by stringent matching of coordinate positions within 10 bp. Due to heterozygosity or low sequencing quality, some loci could not be effectively distinguished and were classified as uncertain. For instance, insertions exceeding 500 bp could not be detected by a single Sanger reaction, and thus were classified as uncertain. After excluding the uncertain sites, the concordances of SV type, length, and breakpoint position between the Sanger sequenced SVs and our high-confidence SVs reached 93.5%, 84.3%, and 70.5%, respectively (Figure 3C). SVs in genic regions displayed higher concordance of type (97.7%) and length (91.9%) than SVs in intergenic regions (89.9% for type and 76.8% for length; Figure 3C). While the concordances of SV type and breakpoint position were not influenced by SV size, the concordance of SV length dropped a little bit as SV size increased (Figure S15). These results suggested that the high concordance from Sanger sequencing highly supported the robustness of our high-confidence SV calls.

### SV benchmark supported by diploid assemblies

With the 8938 high-confidence SV calls, we aimed to construct a benchmark SV callset that could confidently exclude false positives and false negatives of the tested technology in the benchmark regions. To this end, we applied a trio-binning-based approach using the PacBio CCS data to identify haplotype-resolved SVs via haplotype assemblies, acting as another standard for proofing benchmark SVs. Specifically, using 183.6 Gb (61×) short reads of the subject’s father and 184.7 Gb (62×) short reads of the subject’s mother generated by the DNBSEQ-G400 sequencing platform, 77.46% of the subject’s PacBio CCS data were unambiguously partitioned into paternal- and maternal-inherited reads using the trio-binning strategy by integrating five different *k*-mers [26] (Table S2). Then we assembled two haplotypes independently using the biparental CCS reads by Canu [27]. The paternal and maternal haplotype assemblies spanned 2.76 Gb (contig N50 of 726 kb) and 2.92 Gb (contig N50 of 1489 kb), respectively. The haplotype assemblies were aligned against the human reference genome using blasr (v5.3.3) and the SVs were called by smartie-sv independently.

**Figure 4:**
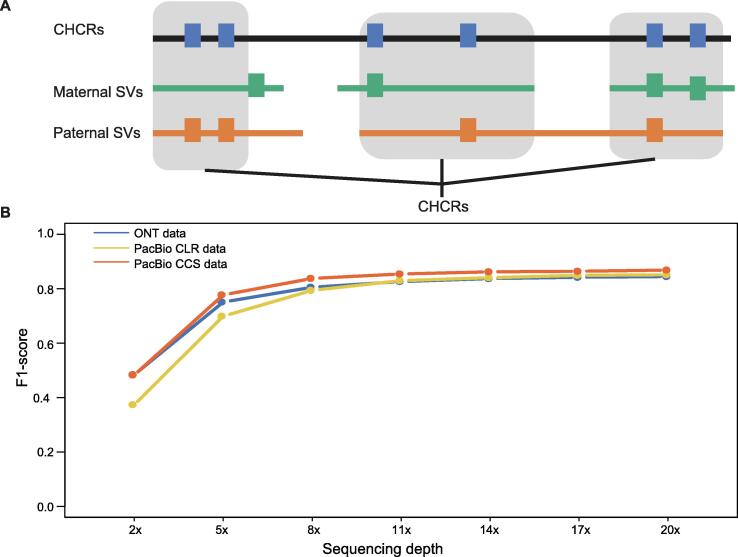
**Establishment and application of the SV benchmark** **A.** Construction of CHCRs. CHCRs were constructed by integrating 8938 high-confidence SVs and SV calls from diploid assemblies. Only high-confidence SVs supported by one or two assemblies were considered as benchmark SVs, and the corresponding genomic regions were defined as CHCRs. **B.** Assessing SV calling performances of different technologies in benchmark regions. Benchmark SVs in CHCRs were used to assess the robustness of three long-read sequencing technologies including PacBio CCS, PacBio CLR, and ONT by calculating their F1-scores under different sequencing depths.

Based on the haplotype assemblies, we constructed the continuous high-confidence regions (CHCRs), on which the identified SVs should be arbitrarily supported by both the high-confidence SV calls and the paternal or maternal, haplotype-resolved SVs (Figure 4A). Finally, we identified 4388 such CHCRs spanning 1.46 Gb with 6882 high-confidence SV calls. These 6882 SV calls constituted our final benchmark SV callset, serving as a gold standard containing comprehensive SVs in benchmark genomic regions in the Reference Material CNGB030001. In other words, in these 4388 benchmark regions, we consider only the 6882 benchmark SVs that are expected in sample CNGB030001. These benchmark SVs can be used to assess the performance of different SV calling platforms and approaches.

### Comparison of the established Asian SV benchmark with the GIAB benchmark

We compared the established Asian benchmark to the recently released GIAB Tier 1 benchmark. Both benchmarks are designed for characterizing deletions and insertions in specified genomic regions. Our Asian benchmark comprises 3346 deletions and 3536 insertions, in contrast to the GIAB benchmark which includes 4069 deletions and 5262 insertions. In total, 3326 SVs (48.3%) were shared by these two benchmarks. Comparison of benchmark SVs is dependent on the identified benchmark genome regions. When comparing the benchmark regions, 1.33 Gb of the 2.51 Gb benchmark regions in GIAB overlapped with our Asian benchmark regions. Within these overlapping regions, 3313 SVs (62.4%) were shared by the two benchmarks, and 1997 (37.6%) and 1785 (35.0%) unique SVs were possessed by our Asian benchmark and GIAB benchmark, respectively (Figure S16). This high overlap supports previous observations that many SVs are shared between different individuals [28]. Compared to common SVs, more small-size deletions and more SINE-Alu insertions were identified in Asian-specific SVs (Figure S17). The unique SVs in the overlapping benchmark regions reflect the genetic diversity of different individuals from different continents (Figure S18), which further highlights the necessity of establishing Asian-specific reference materials and benchmarks.

### Application of the SV benchmark in platform assessment

The SV benchmark enables evaluating the performance of different technologies in SV detection. Here we used our 6882 benchmark SVs to assess the robustness of the three long-read sequencing technologies (PacBio CCS, PacBio CLR, and ONT) by checking their F1-scores under different sequencing depths. F1-scores of all sequencing platforms increased as their sequencing depth increased. When the sequencing depth reached to 11×, all F1-scores approached their saturation points (CCS: 85.4%, CLR: 83.0%, and ONT: 82.6%) (Figure 4B). It should be noted that at a higher sequencing depth (20×), PacBio CCS was the best performer, with a higher F1-score of 86.8% than the other two technologies (CLR: 85.1%, ONT: 84.5%). Our SV benchmark was constructed by integrating SV calls from diverse long-read sequencing platforms and SVs from diploid assemblies. The F1-score results indicate that none of the three platforms could perfectly detect all the SVs in benchmark regions independently. Thus, it is necessary to integrate various approaches and technologies to realize comprehensive and confident SV detection.

We also compared our benchmark SVs to the insertions and deletions revealed by NGS data, which were generated in another parallel project on the same cell line CNGB030001 for evaluating the performance of NGS platforms in SV detection. We evaluated four representative detection tools, including Manta [29], GRIDSS [30], LUMPY [31], and BreakDancer [32] to call SVs from MGISEQ-2000 and NovaSeq 6000 sequencing data, separately. Of the 3536 insertions in our Asian benchmark, 1) 39.5% and 6.7% were detected by Manta and GRIDSS in the MGISEQ data, respectively; 2) 28.3% and 14.3% were detected by Manta and GRIDSS in the NovaSeq data, respectively; and 3) Lumpy and BreakDancer were not capable of detecting insertions from the NGS data (Table S3). Of the 3346 deletions, 1) Manta had 56.8% and 62.8% detection sensitivities in the MGISEQ and NovaSeq data, respectively; 2) GRIDSS, Lumpy, and BreakDancer displayed similar sensitivities in the MGISEQ data (ranging from 38.3% to 41.4%) and in the NovaSeq data (ranging from 30.9% to 50.5%) (Table S3). These results suggested that SV calls from NGS platforms differed among detection tools by displaying different sensitivities and specificities and, in general, showed low sensitivity in detecting insertions. SV detection capacity also differed in different NGS platforms (MGISEQ-2000 *vs*. NovaSeq 6000). These results agree with the fact that different detection strategies emphasize different SV-dependent and library-dependent features, highlighting the need for establishing an SV benchmark using long reads.

## Discussion

A robust SV benchmark provides a gold standard for evaluating the SV detection capacity of diverse strategies and platforms in routine and clinical research. To generate robust high-confidence SV calls, multiple SV callsets from a variety of methods and sequencing technologies need to be evaluated and integrated. PacBio adopts a sequencing-by-synthesis strategy and produces two types of reads. The CLRs emphasize the longest possible reads, and the CCS reads are featured for high accuracy (> 97%). ONT works by monitoring changes in an electrical current when nucleic acids are passed through a protein nanopore. The Bionano linearizes and images long DNA strands that are nicked and fluorescently labeled to produce single-molecule physical maps.

Our work established 8938 high-confidence SV calls by combining SV callsets using four alignment-based SV callers and one *de novo* assembly-based SV caller from the aforementioned state-of-the-art long-read sequencing technologies. We applied experiments and haplotype assemblies for validating and establishing a robust benchmark SV callset. Compared to previous SV work, this study collected high-confidence SV calls by incorporating a deeply sequenced new data type, PacBio CCS, conducted experimental validation, and applied the new trio-binning approach for diploid *de novo* assemblies (combining two parental whole-genome sequencing data) to establish robust benchmark SV calls.

We established an Asian SV benchmark for identifying false negatives and false positives in specified benchmark regions with a well-characterized set of haplotype-resolved SVs. The final benchmark SV callset comprising 6882 SVs is highly robust. First, it was established based on 8938 high-confidence SVs, which were comprehensively constructed by integrating state-of-the-art long-read sequencing technologies. Different sequencing platforms and analysis approaches (alignment-based and assembly-based) complemented each other and their integration was robust in detecting confident SVs. All SVs in the high-confidence set had support from more than one sequencing technology. Second, we validated randomly selected SVs using PCR amplification and Sanger sequencing, confirming the high confidence of our SV calls. Last, we used the trio-binning-based haplotype assemblies to distinguish paternal or maternal SVs. Only haplotype-resolved, high-confidence SVs could be included in the benchmark SV calls. The established Asian benchmark spans 1.46 Gb and covers 6882 SVs supported by at least one diploid haplotype assembly, allowing the community to confidently evaluate the detection capacity for insertions and deletions in future practices.

It should be noted that high accuracy is a prerequisite for the establishment of a benchmark. Therefore, our established benchmark only covered specific regions of the genome with confirmed accuracy, not the whole genome. In these regions, we confirmed that benchmark SVs were highly confident. Our benchmark did not focus on complex SVs (*e.g.*, inversions, duplications, and translocations) either. Importantly, we emphasize that this SV benchmark allows the community to confidently evaluate the performance of various platforms and approaches in detecting insertions and deletions. The deeply sequenced data in this study can be used in future work to extend our understanding of complex SVs. As mentioned above, in another parallel project [33], we generated about 4.16 Tb clean data of the same cell line using seven sequencing strategies in different laboratories, including two BGI regular NGS platforms, three Illumina regular NGS platforms, single tube long fragment read (stLFR) sequencing, and 10X Genomics Chromium linked-read sequencing. These large datasets will provide comprehensive variant information, serving as valuable genomic resources to facilitate future genomic or medical research.

By analyzing extensive SV calls generated by different platforms and calling tools, we found that different technologies had distinct strengths and weaknesses. PacBio CCS detected ∼ 5000 more SVs than PacBio CLR, but neither of them identified the 6 kb LINE insertions using the alignment-based strategy. The CCS assembly-based approach successfully identified four SINE/Alu and LINE elements in insertions and deletions, and detected the largest SV callset with more small-size insertions and deletions. Bionano mapping is based on optical ultra-long single molecules of DNA that are fluorescently labeled at specific restriction sites [24]. Due to its dependency on the density of restriction sites, it failed to accurately detect small-size SVs with the least SVs identified. While other techniques detected more insertions than deletions, ONT was more sensitive to detecting deletions than insertions. It could also effectively detect the 6 kb LINE element insertions.

The established reference material CNGB030001 can serve as an unlimited Asian genomic resource, facilitating future Asian SV and medical studies. EBV transformed cell lines are widely used internationally in routine and clinical research. Usually, cell line genome is relatively stable under a certain number of passages; however, after long-term passages, genomic instability is a common problem in immortalized cell lines, such as tumor cell lines. In our project, genomic instability after long-term passages would not be a concern for our cell line applications. To release as a reference material, we have generated a large quantity of tubes at one time to confirm usage for several years, ensuring low cell passages. A good cell bank management could effectively ensure low cell generations, and regular cell line identifications will help verify the cell line stability. Therefore, cell line CNGB030001 can be widely used for Asian genomic and medical research as a valuable reference material.

## Conclusion

Taking advantage of multiple long-read sequencing platforms, our work established an Asian reference material and developed a robust SV benchmark. PCR amplification and Sanger sequencing validated the high quality of our high-confidence SVs. Trio-binning-based haplotype assemblies were used for identifying the haplotype-resolved SVs to construct the final robust benchmark. The performance of SV calling of different technologies across various sequencing depths provides valuable information for further SV studies. Finally, our established benchmark cell line provides valuable Asian genomic resources for biological and medical research, and the SV benchmark can serve as a gold standard for benchmarking SV detection approaches in clinical practice.

## Materials and methods

### Establishment of immortalized B lymphocyte line

B lymphocyte immortalization was performed according to a published protocol [34] with slight modifications. In brief, 4.5 ml of whole blood was collected from a healthy Chinese donor using a blood collection tube with sodium citrate anticoagulant (Catalog No. 369714, Becton Dickinson, Lake Franklin, NJ). Then, peripheral blood mononuclear cells were isolated from the whole blood by Ficoll density gradient centrifugation (Catalog No. p-05824, GE Healthcare, Chicago, IL). The lymphocytes were simulated and transformed by treating cyclosporin A (Catalog No. 12088, Cayman Chemical, Ann Arbor, MI) and EBV that was prepared by collecting the supernatant of B95-8 cells (ATCC CRL-1612). The performance of lymphocyte transformation was monitored by microscope. After transformation, the immortalized lymphocytes were cultured on a large scale and then divided into 1 × 10^6^ per tube for long-term storage.

### DNA extraction, library preparation, and sequencing

#### DNA extraction for PacBio and ONT

A total of 5 × 10^6^ frozen cells were suspended in 1× PBS buffer to reach a total volume of 2 ml. Then, one volume of ice-cold cell lysis buffer (1.28 M sucrose, 40 mM Tris-HCl, 20 mM MgCl_2_, 4% Triton X-100, pH 7.5) and three volumes of ice-cold distilled water were added. The mixture was incubated for 10 min on ice, and then the nuclear pellets were collected by centrifugation (6000 r/min, 5 min, 4 °C). The nuclei were completely resuspended in extraction buffer (0.8 M guanidine hydrochloride, 30 mM Tris, 30 mM EDTA, 5% Tween-20, 0.5% Triton X-100, pH 8.0) containing 1% sodium dodecyl sulfate (SDS) and proteinase K (2 mg/ml final concentration), and incubated at 56 °C for 2 h. Genomic DNA (gDNA) was extracted by phenol-chloroform-isoamyl alcohol (25:24:1 by volume) and chloroform-isoamyl alcohol (24:1 by volume), and then precipitated with 0.7 volume of isopropyl alcohol at −20 °C for 40 min. The DNA precipitates were washed in ice-cold 80% ethanol twice, collected by centrifugation (12,000 r/min, 15 min, 4 °C), dried under vacuum, and finally resuspended in 100 µl of elution buffer (10 mM Tris-HCl, pH 8.0). To obtain high-quality DNA, an additional purification step was performed right after DNA extraction by using 0.8 volume of magnet beads from Agencourt AMPure XP Kit (Catalog No. A63882, Beckman Coulter, Brea, CA) according to the manufacturer’s instructions. Agilent 4200 Bioanalyzer (Agilent Technologies, Palo Alto, CA) was used to detect the integrity of gDNA. A total of 8 μg gDNA was sheared using g-TUBE (Catalog No. 520079, Covaris, Woburn, MA) and concentrated with the AMPure PB magnetic beads.

#### Library construction and sequencing of PacBio CLR

We used the Pacific Biosciences SMRTbell Template Prep Kit 1.0 to construct each SMRT bell library following the manufacturer’s instructions. The constructed libraries were size-selected on a BluePippin system (Sage Science, Beverly, MA) for molecules ≥ 20 kb, followed by primer annealing and the binding of SMRT bell templates to polymerases using the DNA/Polymerase Binding Kit (Pacific Biosciences, Menlo Park, CA). Finally, sequencing was performed on the Pacific Bioscience Sequel platform (Annoroad Gene Technology, Beijing, China) for 10 h by CLR mode with the Sequel System (Pacific Biosciences).

#### Library construction and sequencing of PacBio CCS

SMRT bell libraries were prepared using the ‘Express Template Prep Kit 1.0’ protocol (Pacific Biosciences). A total of 5 µg gDNA was sheared to ∼ 15 kb fragments using g-TUBE (Catalog No. 520079, Covaris) plus centrifugation (2000 *g*, 2 min, twice). The fragments ware size-selected for 10 kb using the BluePippin system (Sage Science) by marker (0.75% DF Marker S1 High-Pass 6-10 kb vs3) for the 10–20 kb DNA target fragments. Quality control of the libraries was performed by Qubit fluorometer (Life Technologies, Carlsbad, CA) and Bioanalyzer 2100 (Agilent Technologies). The prepared library was loaded into SMRT cell 1M by Sequel Binding Kit 3.0 (Pacific Biosciences) and finally sequenced by CCS mode with the Sequel System (Pacific Biosciences).

#### Library construction and sequencing of ONT

gDNA libraries were prepared using the Ligation Sequencing 1D Kit (Catalog No. SQK-LSK109, Oxford Nanopore Technologies, Oxford, UK). End-repair and dA-tailing of DNA fragments were performed using the Ultra II End Repair/dA-Tailing Module (Catalog No. E7546, New England Biolabs, Ipswich, MA) following the manufacturer’s recommendations. The dA-tailed sample was tethered to 1D adapter by Quick Ligation Module (Catalog No. E6056, New England Biolabs). Finally, the prepared DNA library was loaded into FLO-PRO002 Flow Cell and sequenced on PromethION (Oxford Nanopore Technologies).

#### DNA extraction and sequencing for Bionano

The isolation of high-molecular-weight gDNA from immortalized B lymphocyte line was performed with the Bionano Prep Cell Culture DNA Isolation Kit (Catalog No. 80004, Bionano Genomics, San Diego, CA) according to the standard protocol of Bionano Prep Cell Culture DNA Isolation Protocol (Document No.: 30026). Sequence-specific labeling of megabase gDNA for Bionano mapping was conducted by nicking, labeling, repairing, and staining (NLRS) following the standard protocol of Bionano Prep Labeling-NLRS. The labeled gDNA was transferred into Bionano Genomics Saphyr (San Diego, CA) for scanning to obtain the optical map.

### SV calling based on different platforms and methods

#### Alignment-based SV calling

For CLRs, BAM files of CLRs were exported from SMRT Link (v6.0.0.47841), and aligned to the reference genome (hs37d5) using minimap2 (v2.15-r906-dirty) [35] with the following parameters: -x map-pb -a --eqx -L -O 5,56 -E 4,1 -B 5 –secondary = no -z 400,50 -r 2k -Y -R “@RG\tID:rg1a\tSM:human”.

For CCS reads, BAM files were aligned to the reference genome (hs37d5) using minimap2 (v2.15-r906-dirty) [35] with the parameters “-R -t 2 --MD -Y -L -a -x map-pb”. According to the mapping positions, SAMtools (v0.1.19) [36] was used to sort the alignments with default parameters. To identify SVs, pbsv (v2.1.1) [37] with default parameters was used to sort alignment files.

For ONT reads, reads with quality score > 7 were aligned to the reference genome (hs37d5) using ngmlr (v0.2.7) [38] with the parameter “--presets nanopore”. SVs were called using sniffles (version 1.0.8) [38] with the parameters “--min_support 1 --threads 8 --num_reads_report -1 –genotype”.

For Bionano data, Bionano data were generated from the enzyme *Bsp*QI, and SVs were called using Bionano Solve pipeline (v3.1) [39] with default parameters.

#### De novo assembly-based SV calling

Falcon (v0.3.0) [40] was used for assembly, and contigs were aligned to the reference genome (hs37d5) using blasr (v5.3.3) [41]. SV calling was performed with smartie-sv [25], [42].

### Integration of the high-confidence SV calls

The high-confidence SV calls were integrated from all candidate SV callsets by the following steps: 1) the same type of SVs within 1 kb with sequence change < 20% were merged into a single SV using SVmerge (v1.2r27) [43]; 2) SVs located in centromeres, telomeres, segmental duplications, and short tandem repeat regions were removed according to the SV annotations by ANNOVAR (v20160201) [44]; 3) SVs that were detected by PacBio CCS and supported by either PacBio CLR or ONT were retained, and SVs that were longer than 1.5 kb and supported by PacBio CCS assembly and Bionano mapping were also retained; 4) Hawkeye (v2.0) [45] was used for SV visualization by automatically outputting images for manual checking.

### SV validation by PCR amplification and Sanger sequencing

We performed validation for two batches of randomly selected SVs. A PCR amplification was considered successful if a clear single band was observed or the expected size band could be purified and separated by gel cutting; conversely, failed SVs showed ambiguous bands. To evaluate the effect of primer design, failed SVs from the first batch were repeated for PCR amplification in the second batch. The corresponding primers were designed with Primer3 by default parameters [46]. Amplification results for each amplicon were validated by electrophoresis, and the products were loaded onto 3730 sequencers with the paired-end sequencing mode (ThermoFisher Scientific, Waltham, MA). Raw sequencing results were analyzed by Sequence Scanner Software v2.0, and the low-quality parts were trimmed. The clean reads were mapped to the reference genome hs37d5 by BLAST, and the mapping results were manually checked for SVs. For manual curation, the following criteria were used to evaluate the accuracy of previous SV calls: 1) if there was an SV event supported by any single Sanger read; 2) if a previously called SV could match a Sanger call within a 10 bp difference in size; and 3) if the breakpoint of a previously called SV could match that of a Sanger call within a 10 bp difference.

### Construction of diploid haplotype genomes using trio-binning

Short reads from the parents were used to identify *k*-mers unique to each parent and partition (“trio-binning”) the CCS reads. The trio-binning pipeline was applied to partition paternal and maternal CCS reads [26], [47] using five different *k*-mers, including 21 bp (previously reported for trio-binning) and longer *k*-mers of 41 bp, 51 bp, 61 bp, and 81 bp. To realize accurate partition, one integration method was used following two criteria: for one CCS read, 1) at least two different *k*-mers support the same parental source; and 2) more than half of the different *k*-mers support the same parental source.

To obtain paternal and maternal haplotype genomes, we used Canu (v1.8-r9528) [27] to assemble paternal and maternal CCS reads with the parameters “-trim-assemble genomeSize = 3100m correctedErrorRate = 0.039 -pacbio-corrected”. Meanwhile, unassigned CCS reads were used in both assemblies. Lastly, the paternal and maternal haplotype assemblies were aligned against the human reference genome using blasr (v5.3.3), and SVs were called by smartie-sv independently.

### SV benchmark construction, comparison, and application

To establish the benchmark SV callset, we identified the CHCRs by combining 8938 high-confidence SVs and SVs called from diploid assemblies. Only high-confidence SVs supported by one or two assemblies were retained in the benchmark SV callset. To evaluate the capability of different platforms, SV calls detected by different technologies were all converted into VCF formats and evaluated against the CHCRs using Truvari (v1.3) [48] with default parameters. To compare our Asian benchmark to the GIAB benchmark, we used SVmerge (v1.2r27) with the default parameters “-d 1000” for comparing breakpoint positions, “-l 0.5” for comparing SV length difference, and “-r 0.5” for checking SV overlap. Overlapping and unique SVs were enriched for gene pathways by R package clusterProfiler.

SVs from two NGS platforms (MGISEQ-2000 and NovaSeq 6000) which sequenced the same cell line, were called using four tools and the hs37d5 reference genome with the following parameters: GRIDSS (default parameters), LUMPY (default parameters), Manta (minCandidateSpanningCount = 3, minScoredVariantSize = 50, minDiploidVariantScore = 10, minPassDiploidVariantScore = 20, minPassDiploidGTScore = 15, minSomaticScore = 10, minPassSomaticScore = 30, useOverlapPairEvidence = 0, enableRemoteReadRetrievalForInsertionsInGermlineCallingModes = 1, enableRemoteReadRetrievalForInsertionsInCancerCallingModes = 0), and BreakDancer (num:10001, lower:78.10, upper:465.35, mean:254.28, std:48.32, SWnormality:-31.28). For each software, triplicate SV calls were made and then integrated into a final call by SURVIVOR using the following parameters: 1000 2 1 1 0 30. To find common SVs between NGS SV calls and our SV benchmark, we used SVmerge (v1.2r27) with default parameters as above.

## Ethical statement

The written informed consent was obtained from the participating subject. The experimental procedures were in accordance with the guidelines approved by the institutional review board on bioethics and biosafety of BGI (IRB-BGI). The experiment was authorized by IRB-BGI (under No. FT19038), and the review procedures in IRB-BGI meet good clinical practice (GCP) principles.

## Data availability

The raw sequence data in this study have been deposited in the Genome Sequence Archive [49] at the National Genomics Data Center, Beijing Institute of Genomics, Chinese Academy of Sciences / China National Center for Bioinformation (GAS: HRA000324), and are publicly accessible at https://ngdc.cncb.ac.cn/gsa-human/browse/HRA000324.

## CRediT author statement

**Xiao Du:** Writing - original draft, Writing - review & editing. **Lili Li:** Investigation, Resources, Formal analysis. **Fan Liang:** Formal analysis, Software, Supervision. **Sanyang Liu:** Formal analysis, Software. **Wenxin Zhang:** Resources. **Shuai Sun:** Formal analysis, Writing - original draft. **Yuhui Sun:** Formal analysis. **Fei Fan:** Investigation, Validation. **Linying Wang:** Investigation, Validation. **Xinming Liang:** Methodology, Data curation, Formal analysis. **Weijin Qiu:** Software, Formal analysis, Data curation. **Guangyi Fan:** Writing - original draft, Supervision. **Ou Wang:** Supervision, Methodology, Investigation, Validation. **Weifei Yang:** Formal analysis. **Jiezhong Zhang:** Resources, Formal analysis. **Yuhui Xiao:** Visualization, Software, Formal analysis. **Yang Wang:** Visualization, Software, Formal analysis. **Depeng Wang:** Conceptualization, Supervision, Project administration. **Shoufang Qu:** Supervision, Investigation, Validation. **Fang Chen:** Conceptualization, Resources, Investigation, Validation. **Jie Huang:** Conceptualization, Resources, Supervision, Project administration, Funding acquisition. All authors have read and approved the final manuscript.

## Competing interests

The authors have declared no competing interests.

## Supplementary material

Supplementary Figure S1 **The length and GC content distributions of PacBio CLR sequencing reads** Read length (bp) and GC content (%) were plotted on the scatter plot. Histograms displayed read numbers. CLR, continuous long read.

Supplementary Figure S2 **Read quality of PacBio CCS** The quality of reads varies with the number of PacBio CCS passes. CCS, circular consensus sequencing.

Supplementary Figure S3 **The length and GC content distributions of PacBio CCS sequencing reads** Read length (bp) and GC content (%) were plotted on the scatter plot. Histograms displayed read numbers. CCS, circular consensus sequencing.

Supplementary Figure S4 **The length and GC content distributions of Oxford Nanopore reads** Read length (bp) and GC content (%) were plotted on the scatter plot. Histograms displayed read numbers.

Supplementary Figure S5 **The size distributions for candidate deletions and insertions from PacBio CLR** Distributions displayed 300 bp and 6kb peaks related to SINE-Alu and LINE elements, respectively. CLR, continuous long read.

Supplementary Figure S6 **The size distributions for candidate deletions and insertions from PacBio CCS** Distributions displayed 300 bp and 6kb peaks related to SINE-Alu and LINE elements, respectively. CCS, circular consensus sequencing

Supplementary Figure S7 **The size distributions for candidate deletions and insertions from Oxford Nanopore** Distributions displayed 300 bp and 6kb peaks related to SINE-Alu and LINE elements, respectively.

Supplementary Figure S8 **The size distributions for candidate deletions and insertions from Bionano** Distributions displayed 6kb peaks related to LINE elements in deletions.

Supplementary Figure S9 **Synteny between PacBio CCS contigs and reference genome** Dot plot showed the synteny between assembled contigs from PacBio CCS reads and the hs37d5 reference genome. CCS, circular consensus sequencing

Supplementary Figure S10 **The size distributions for candidate deletions and insertions from PacBio CCS assembly** Distributions displayed 300 bp and 6kb peaks related to SINE-Alu and LINE elements, respectively. CCS, circular consensus sequencing

Supplementary Figure S11 **The relationship between chromosome length and SV counts across the autosome chromosomes** SV counts displayed a good linear correlation with the chromosome length (R^2^ = 0.85, *p*-value < 0.0001).

Supplementary Figure S12 **The counts of unique SVs and SV overlapping among different candidate SV callsets** PBAS_SV, assembly-based SV calls; BN_SV, Bionano SV calls; ONT_SV, Oxford Nanopore SV calls; PBSB_SV, PacBio CLR SV calls; PBHF_SV, PacBio CCS SV calls.

Supplementary Figure S13 **Distribution of PCR Amplification results across different SV lengths** Blue and red histograms refer to successful and failed amplifications, respectively. Green line represents the PCR success rate.

Supplementary Figure S14 **Consistency rates of SV type, SV length, and breakpoint position between Sanger sequenced SVs and high-confidence SVs** Uncertain sites were not excluded. Blue, red, and gray histograms refer to accordance, discordance, and uncertain sites, respectively. Green line represents the consistency rate.

Supplementary Figure S15 **Consistency rates of SV type, SV length, and SV position between Sanger sequenced SVs and high-confidence SVs across different SV lengths** Uncertain sites were not excluded. Dark blue, blue, and red lines represent consistency rates of SV type, SV length, and SV position, respectively.

Supplementary Figure S16 **Comparison of the Asian CNGB030001 benchmark and the GIAB HG002 benchmark within the overlapping benchmark regions** The overlapping benchmark regions spanned 1.33 Gb on the genome. Both unique and overlapping SVs were identified.

Supplementary Figure S17 **Size distributions for common and Asian unique SVs** The Asian CNGB030001 benchmark was compared to the GIAB HG002 benchmark within their overlapping benchmark regions.

Supplementary Figure S18 **GO enrichment of Asian-benchmark-specific SVs** The Asian CNGB030001 benchmark was compared to the GIAB HG002 benchmark within their overlapping benchmark regions for identifying Asian-benchmark-specific SVs.

Supplementary Table S1 **Counts of high-confidence SVs on each chromosome**

Supplementary Table S2 **Trio binning of CCS reads**

Supplementary Table S3 **Comparison between SVs from NGS platforms and benchmark SVs**
