# Supplementary figures and images for: Robust Benchmark Structural Variant Calls of An Asian Using State-of-the-art Long-read Sequencing Technologies
Source: Genomics Proteomics Bioinformatics. 2021 Mar 2;20(1):192–204. doi: 10.1016/j.gpb.2020.10.006 (PMC9510867; doi:10.1016/j.gpb.2020.10.006)

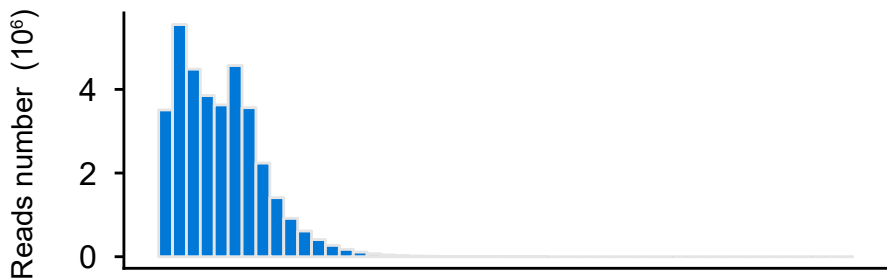

Reads number

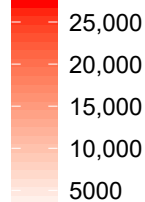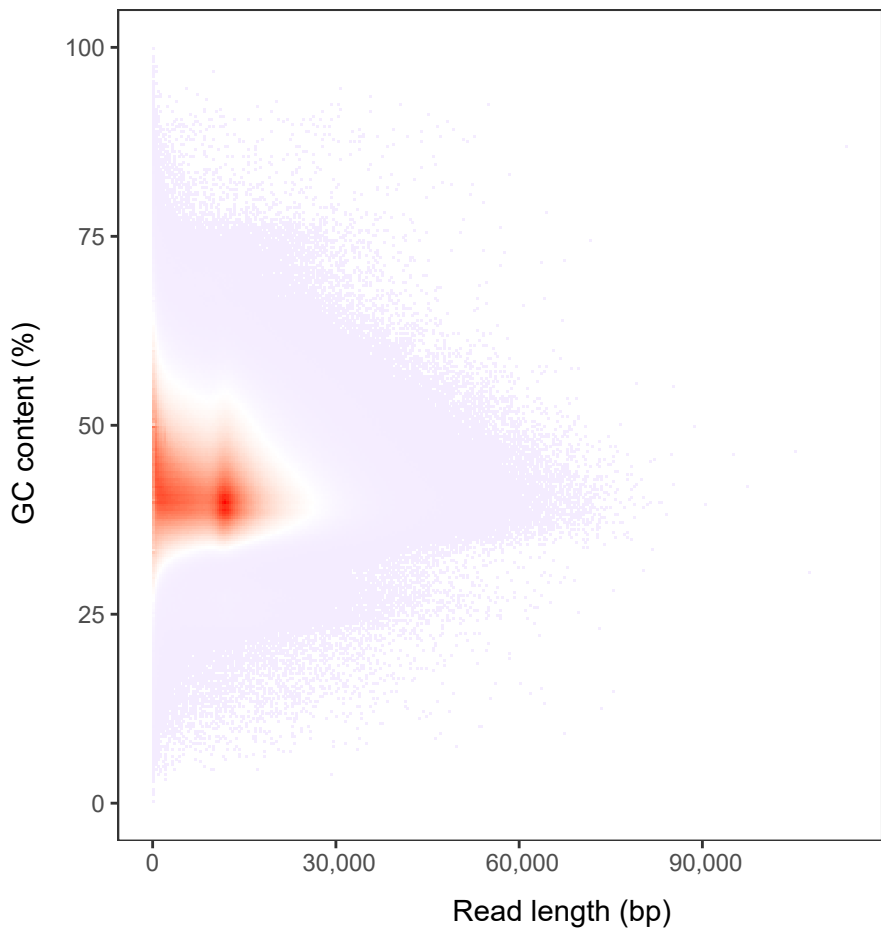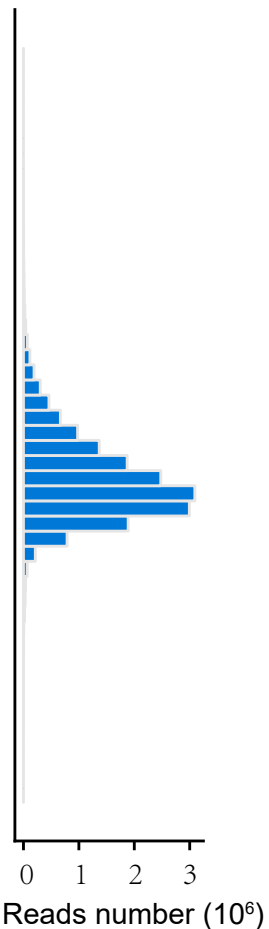

Supplement: Supplementary Figure S1 — The length and GC content distributions of PacBio CLR sequencing reads Read length (bp) and GC content (%) were plotted on the scatter plot. Histograms displayed read numbers. CLR, continuous long read. [file mmc1.pdf]

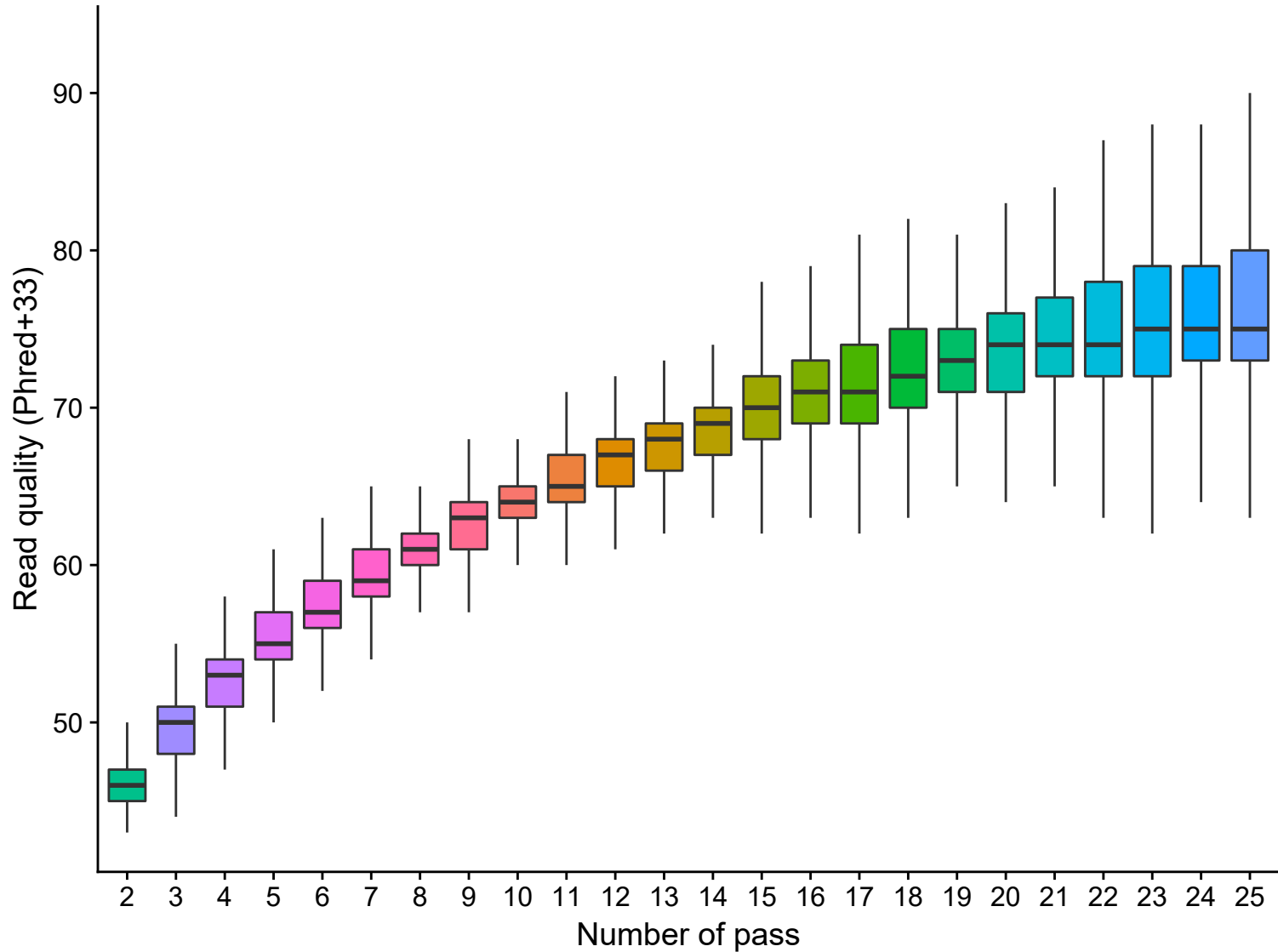

Supplement: Supplementary Figure S2 — Read quality of PacBio CCS The quality of reads varies with the number of PacBio CCS passes. CCS, circular consensus sequencing. [file mmc2.pdf]

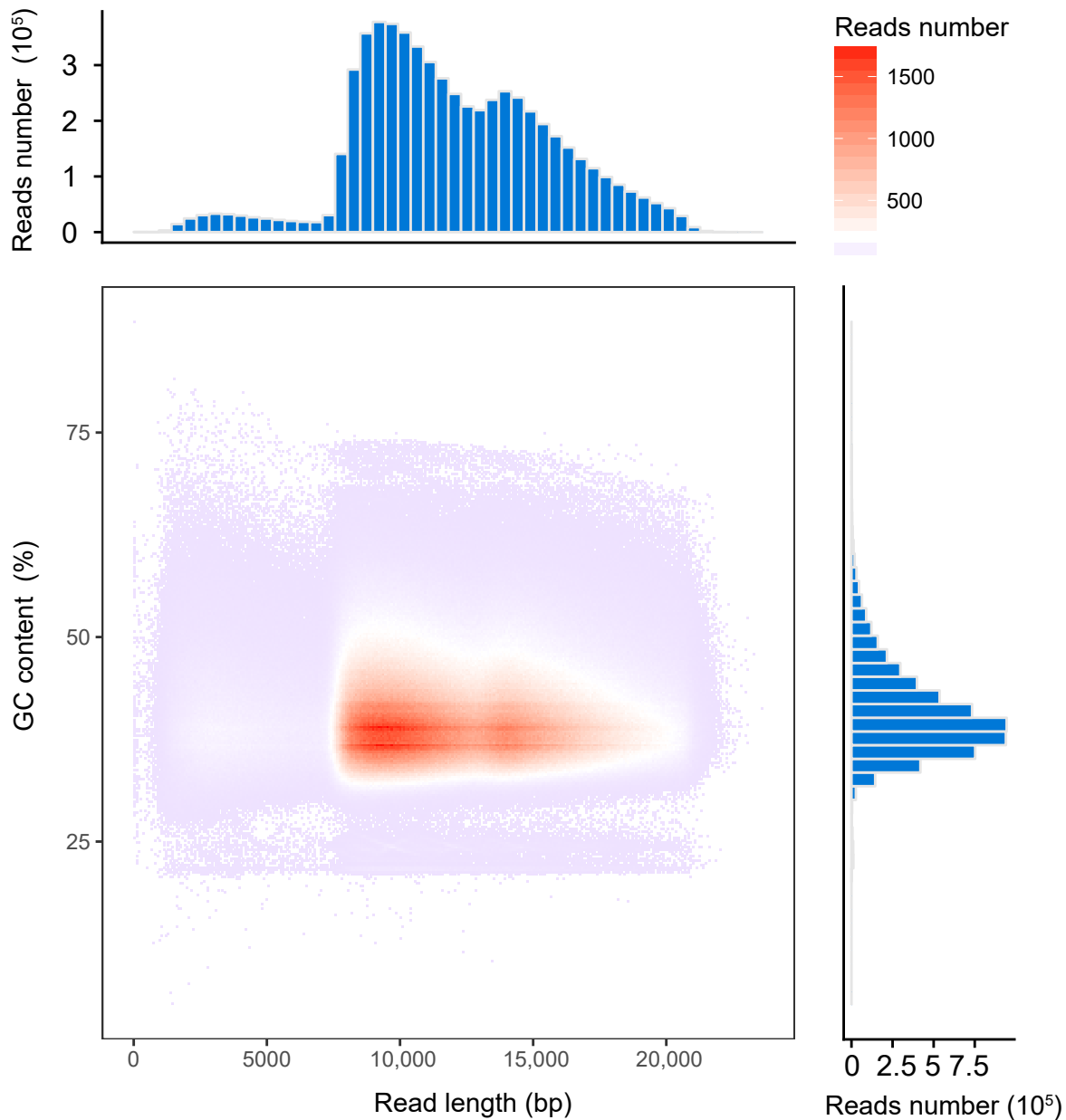

Supplement: Supplementary Figure S3 — The length and GC content distributions of PacBio CCS sequencing reads Read length (bp) and GC content (%) were plotted on the scatter plot. Histograms displayed read numbers. CCS, circular consensus sequencing. [file mmc3.pdf]

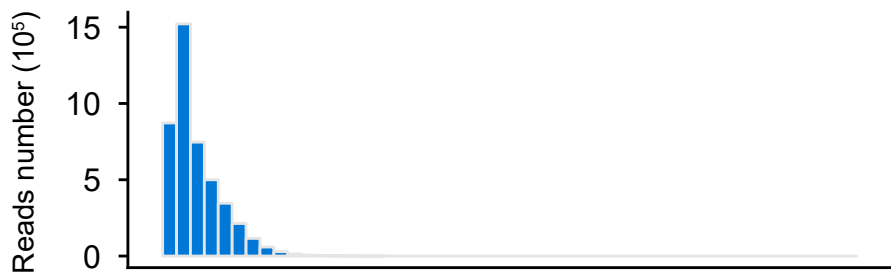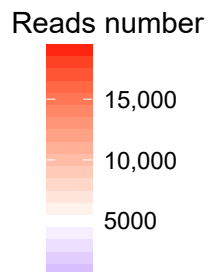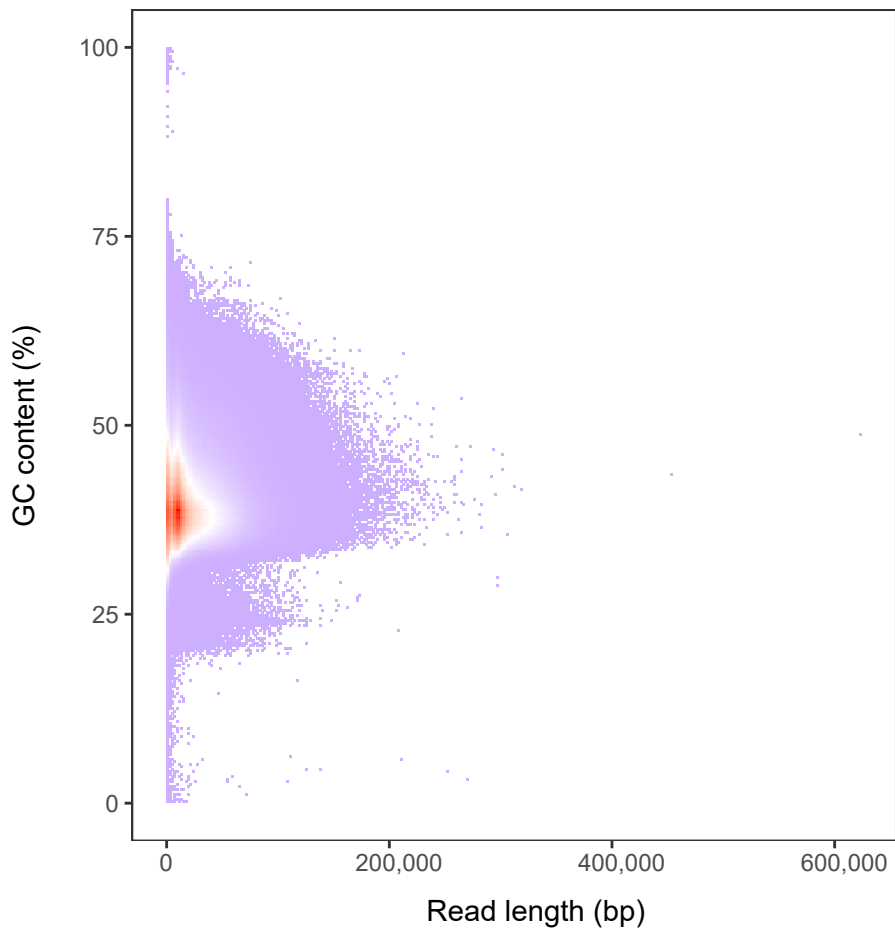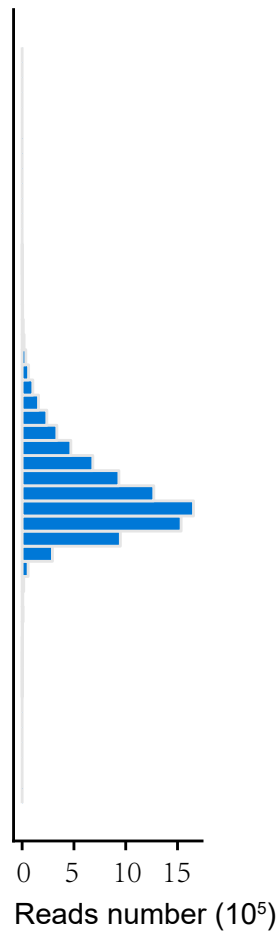

Supplement: Supplementary Figure S4 — The length and GC content distributions of Oxford Nanopore reads Read length (bp) and GC content (%) were plotted on the scatter plot. Histograms displayed read numbers. [file mmc4.pdf]

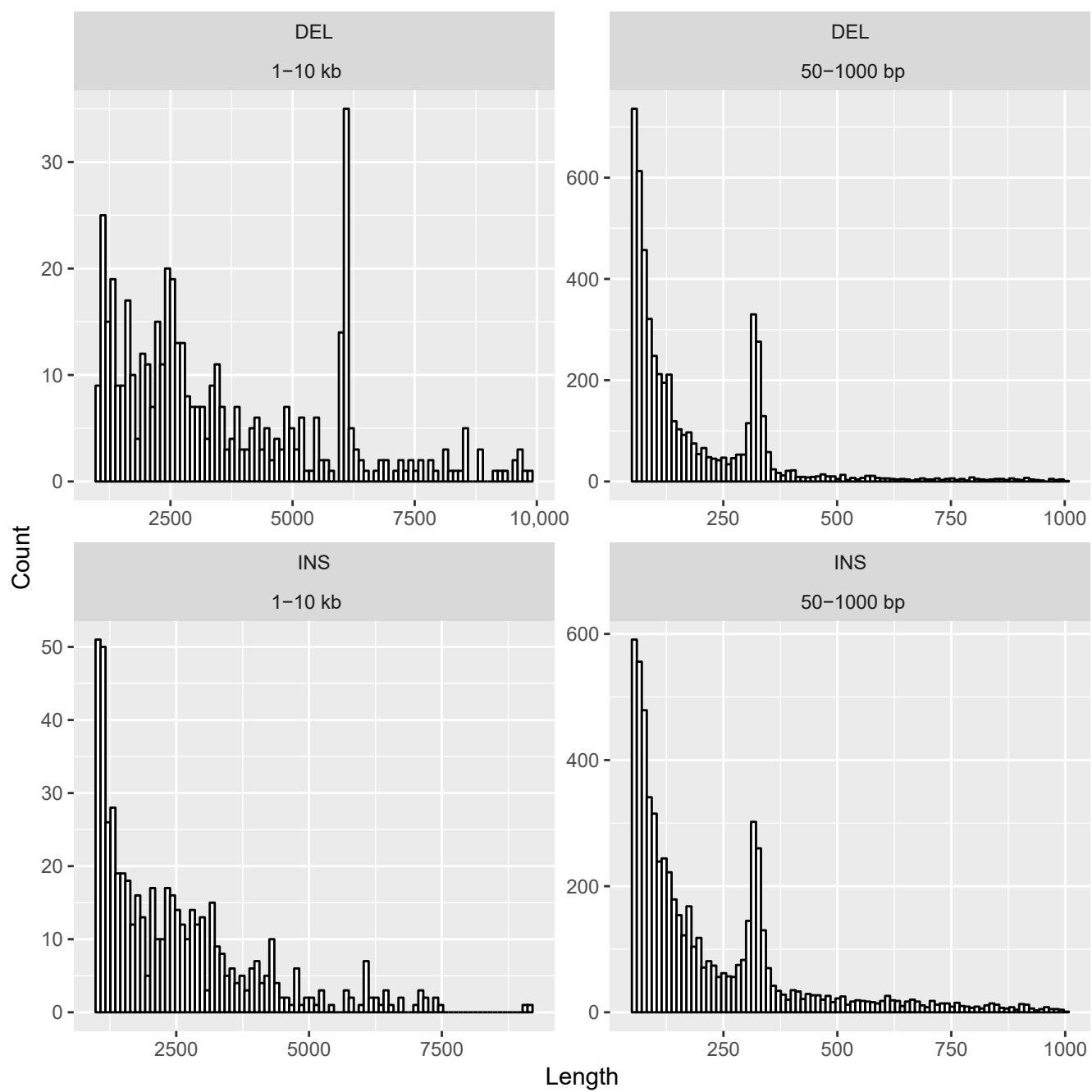

Supplement: Supplementary Figure S5 — The size distributions for candidate deletions and insertions from PacBio CLR Distributions displayed 300 bp and 6kb peaks related to SINE-Alu and LINE elements, respectively. CLR, continuous long read. [file mmc5.pdf]

Count

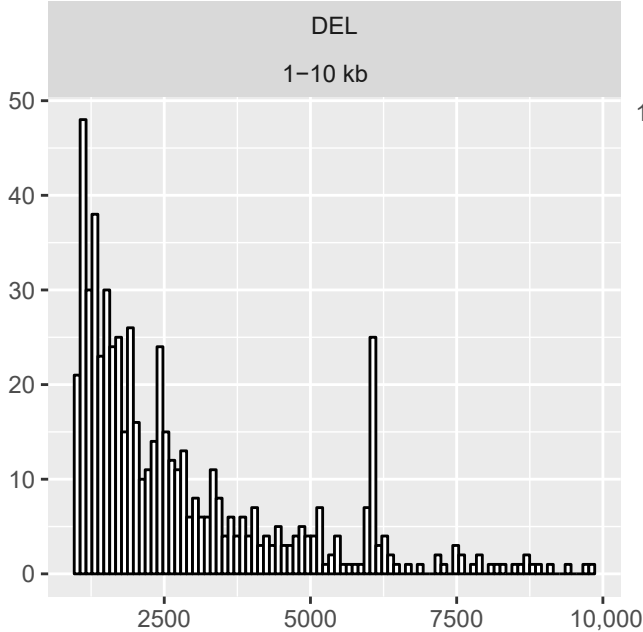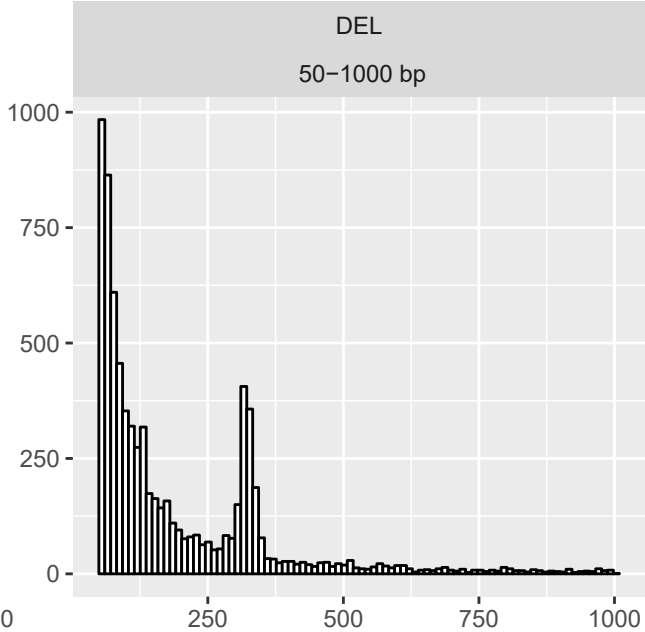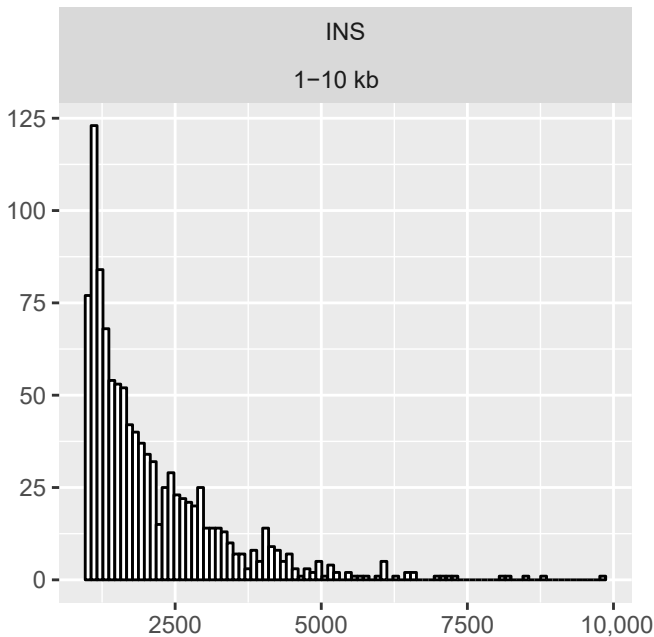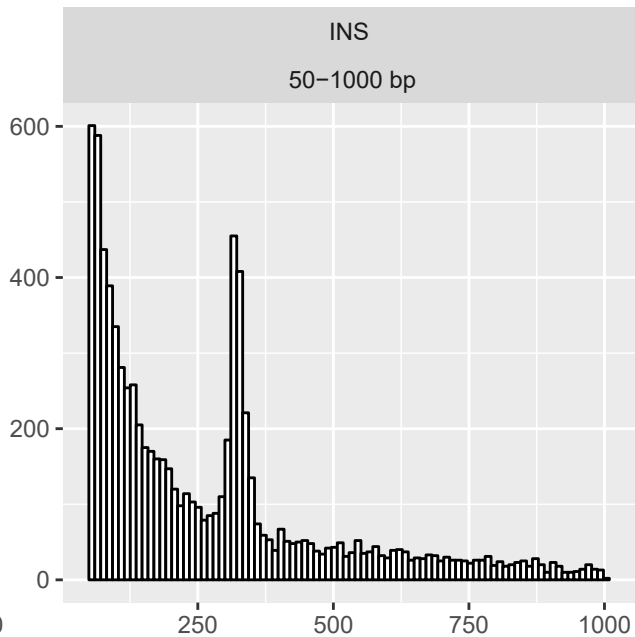

Length

Supplement: Supplementary Figure S6 — The size distributions for candidate deletions and insertions from PacBio CCS Distributions displayed 300 bp and 6kb peaks related to SINE-Alu and LINE elements, respectively. CCS, circular consensus sequencing [file mmc6.pdf]

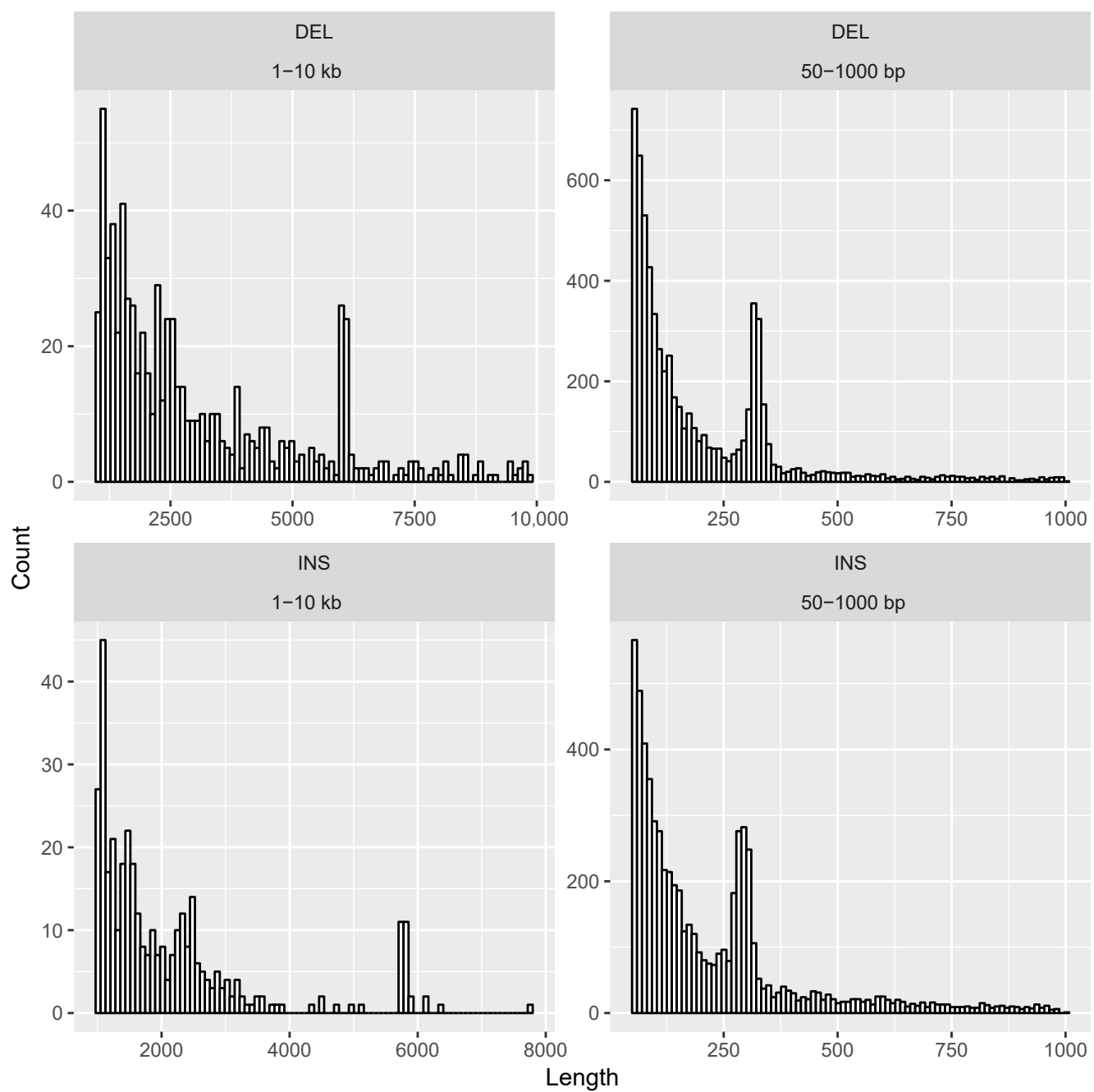

Supplement: Supplementary Figure S7 — The size distributions for candidate deletions and insertions from Oxford Nanopore Distributions displayed 300 bp and 6kb peaks related to SINE-Alu and LINE elements, respectively. [file mmc7.pdf]

Count

DEL

1–10 kb

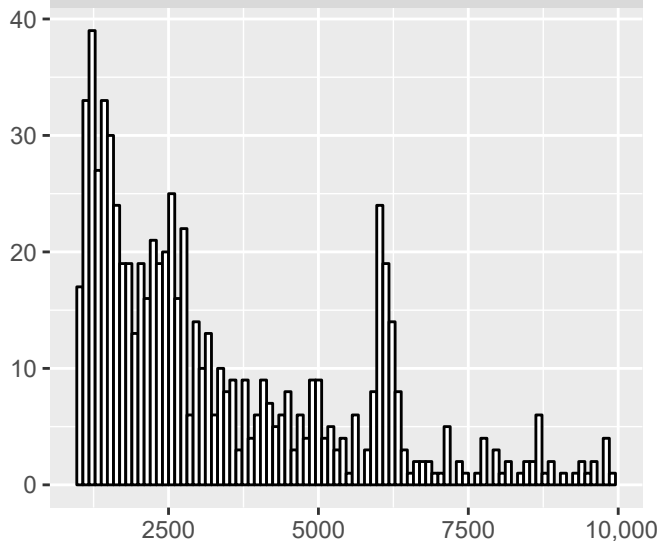

DEL

50–1000 bp

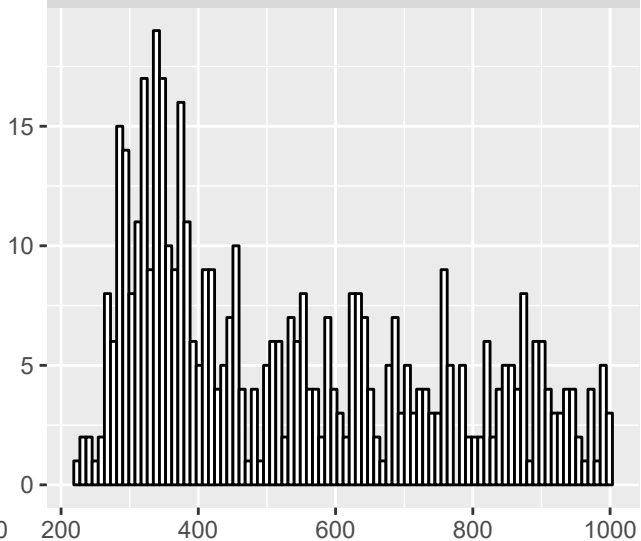

INS

1–10 kb

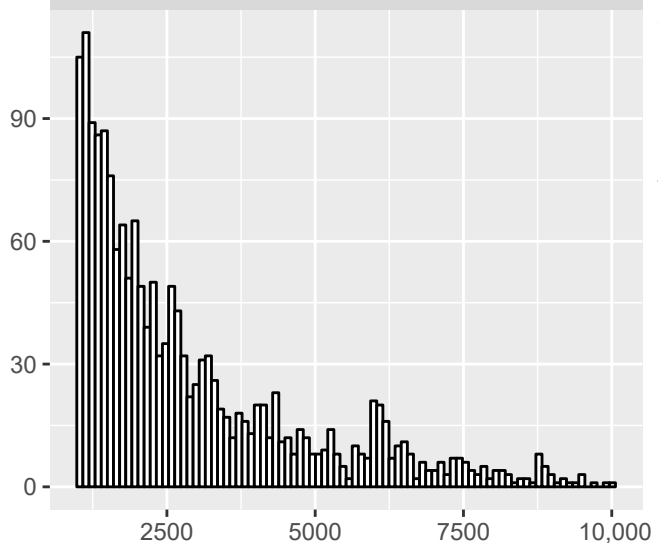

INS

50–1000 bp

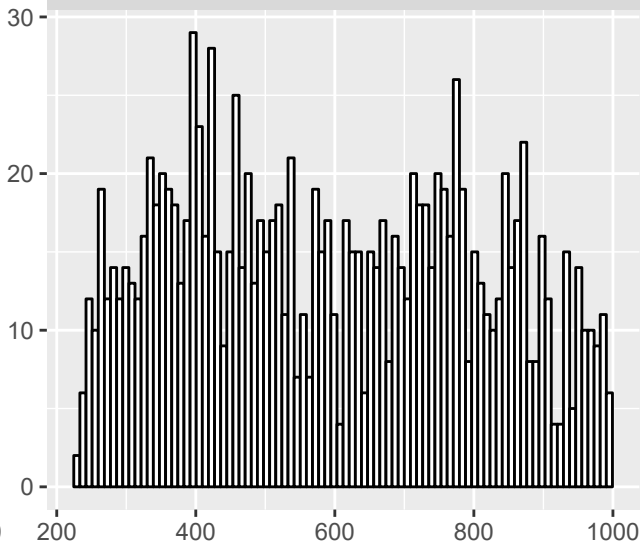

Length

Supplement: Supplementary Figure S8 — The size distributions for candidate deletions and insertions from Bionano Distributions displayed 6kb peaks related to LINE elements in deletions. [file mmc8.pdf]

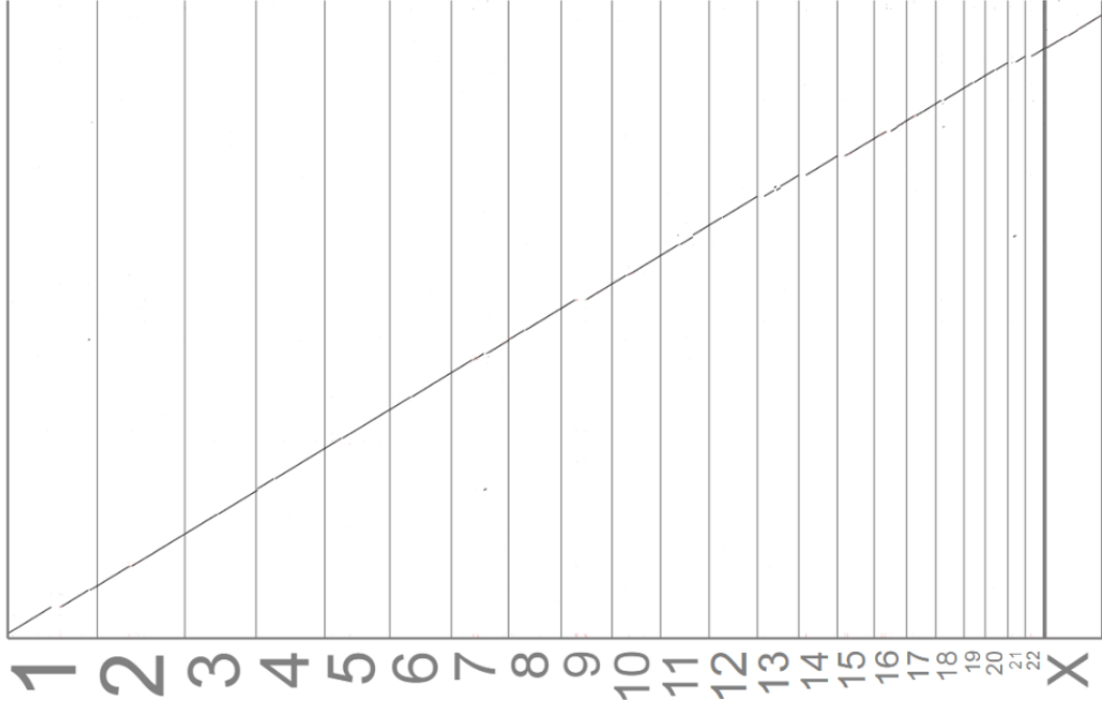

Supplement: Supplementary Figure S9 — Synteny between PacBio CCS contigs and reference genome Dot plot showed the synteny between assembled contigs from PacBio CCS reads and the hs37d5 reference genome. CCS, circular consensus sequencing [file mmc9.pdf]

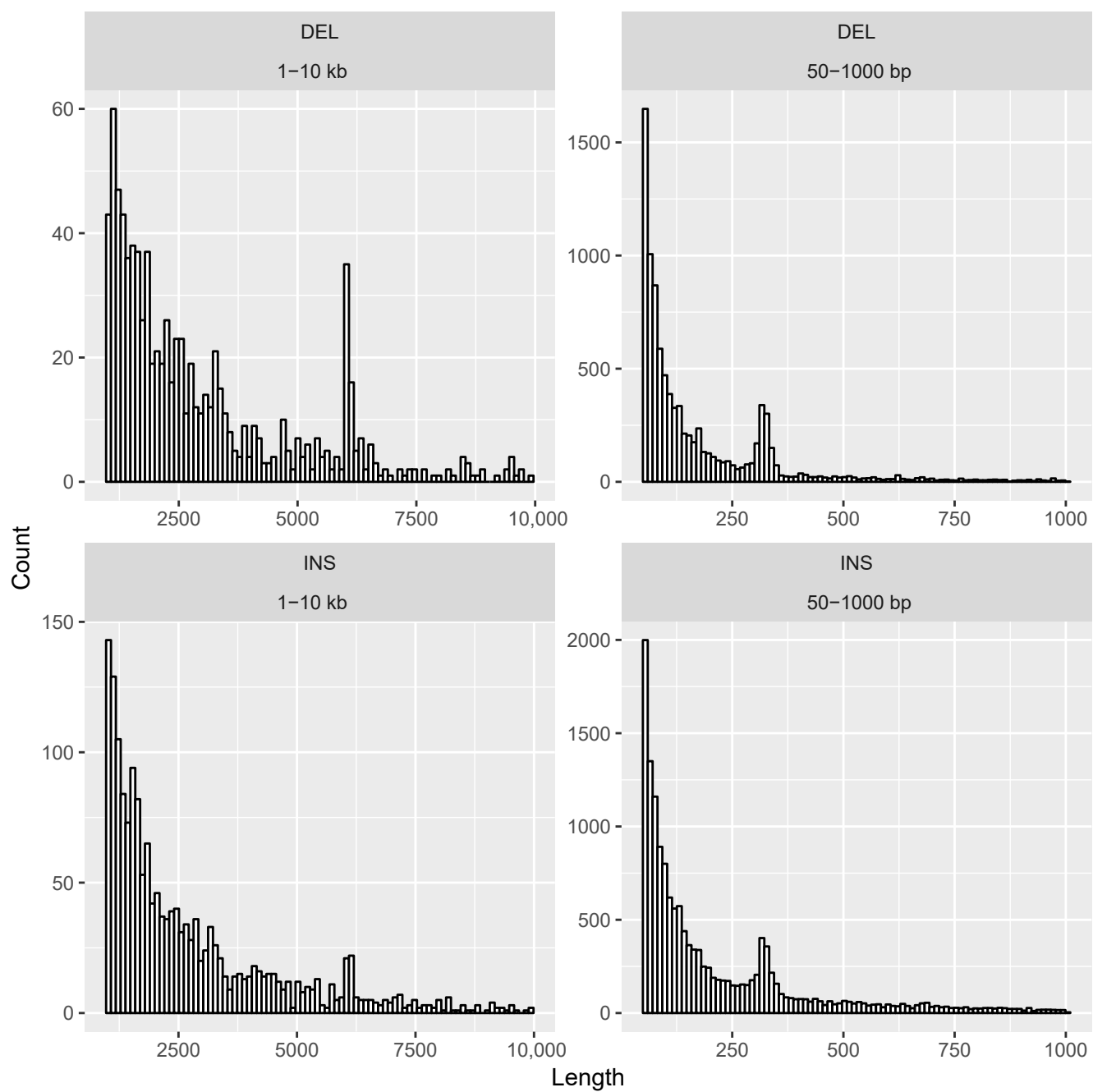

Supplement: Supplementary Figure S10 — The size distributions for candidate deletions and insertions from PacBio CCS assembly Distributions displayed 300 bp and 6kb peaks related to SINE-Alu and LINE elements, respectively. CCS, circular consensus sequencing [file mmc10.pdf]

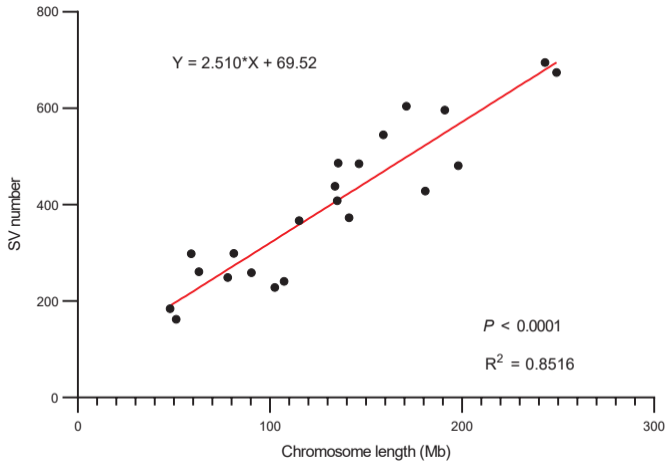

Supplement: Supplementary Figure S11 — The relationship between chromosome length and SV counts across the autosome chromosomes SV counts displayed a good linear correlation with the chromosome length (R2 = 0.85, p-value < 0.0001). [file mmc11.pdf]

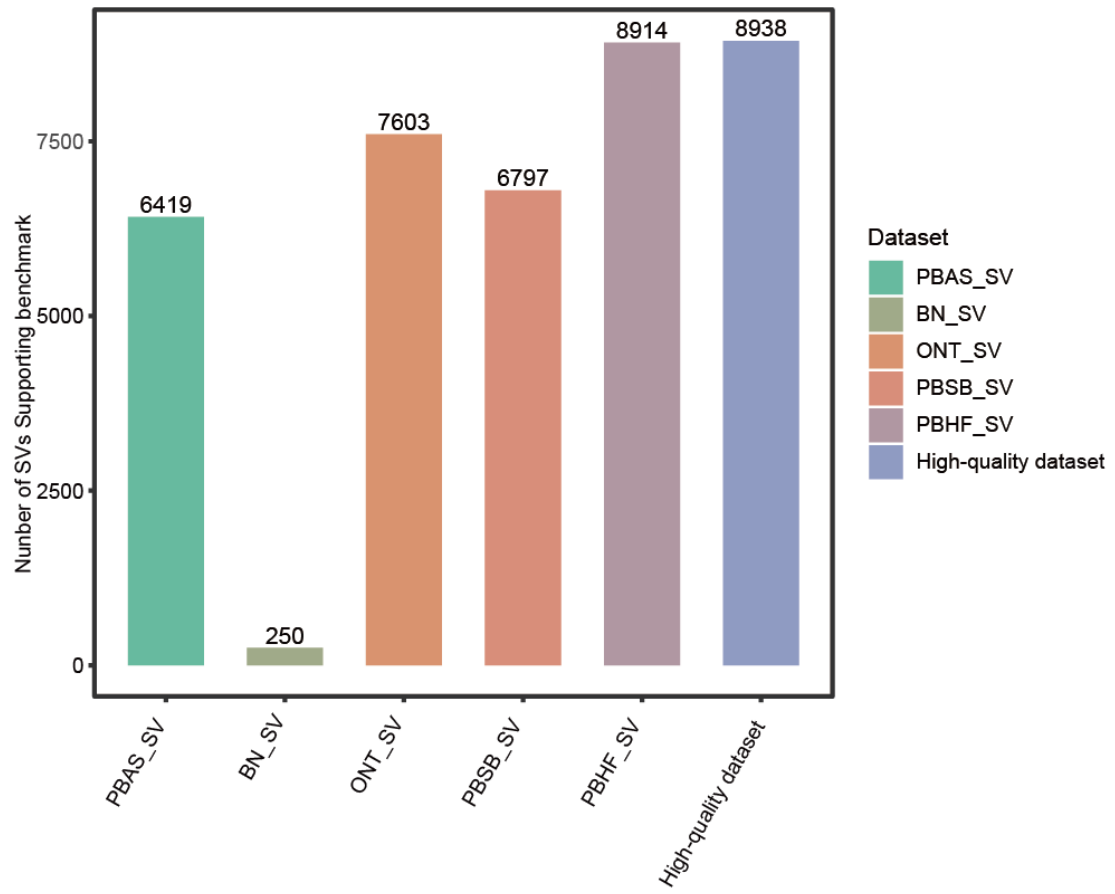

Supplement: Supplementary Figure S12 — The counts of unique SVs and SV overlapping among different candidate SV callsets PBAS_SV, assembly-based SV calls; BN_SV, Bionano SV calls; ONT_SV, Oxford Nanopore SV calls; PBSB_SV, PacBio CLR SV calls; PBHF_SV, PacBio CCS SV calls. [file mmc12.pdf]

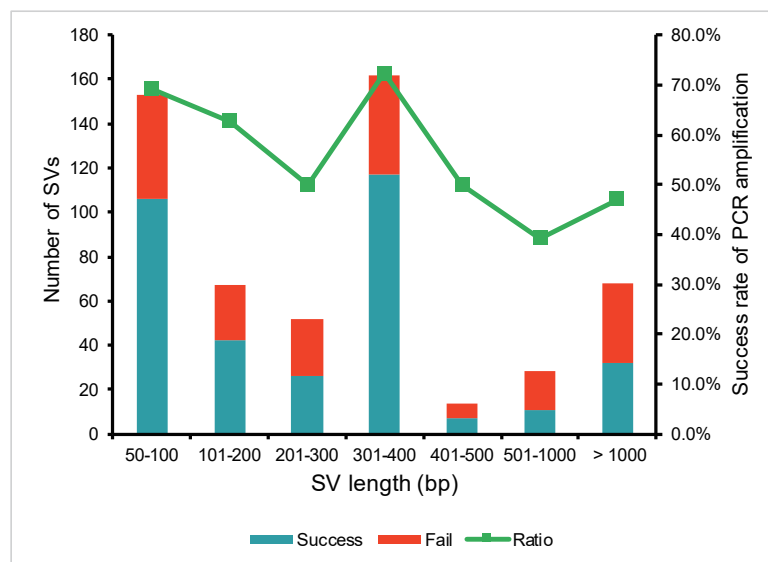

Supplement: Supplementary Figure S13 — Distribution of PCR Amplification results across different SV lengths Blue and red histograms refer to successful and failed amplifications, respectively. Green line represents the PCR success rate. [file mmc13.pdf]

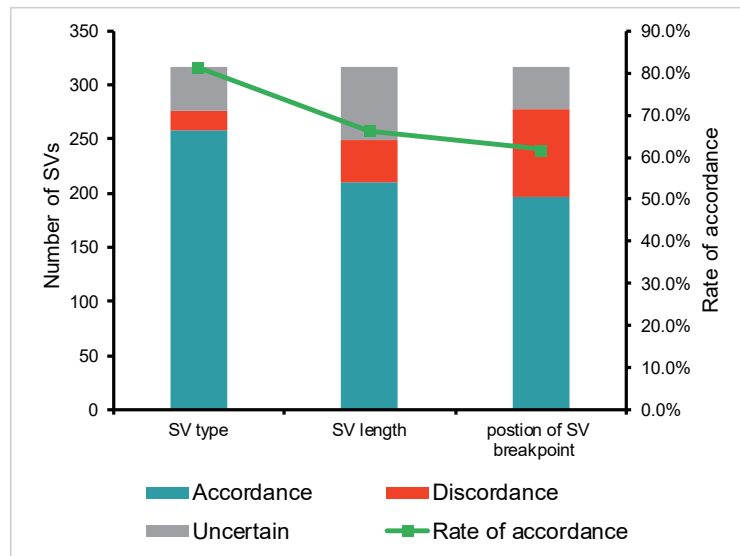

Supplement: Supplementary Figure S14 — Consistency rates of SV type, SV length, and breakpoint position between Sanger sequenced SVs and high-confidence SVs Uncertain sites were not excluded. Blue, red, and gray histograms refer to accordance, discordance, and uncertain sites, respectively. Green line represents the consistency rate. [file mmc14.pdf]

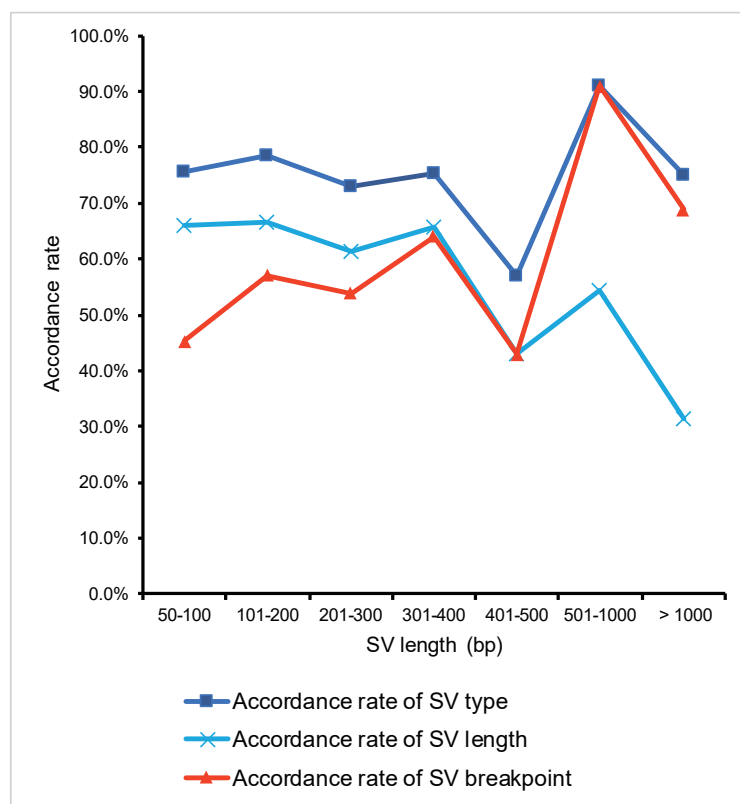

Supplement: Supplementary Figure S15 — Consistency rates of SV type, SV length, and SV position between Sanger sequenced SVs and high-confidence SVs across different SV lengths Uncertain sites were not excluded. Dark blue, blue, and red lines represent consistency rates of SV type, SV length, and SV position, respectively. [file mmc15.pdf]

CNGB030001

HG002

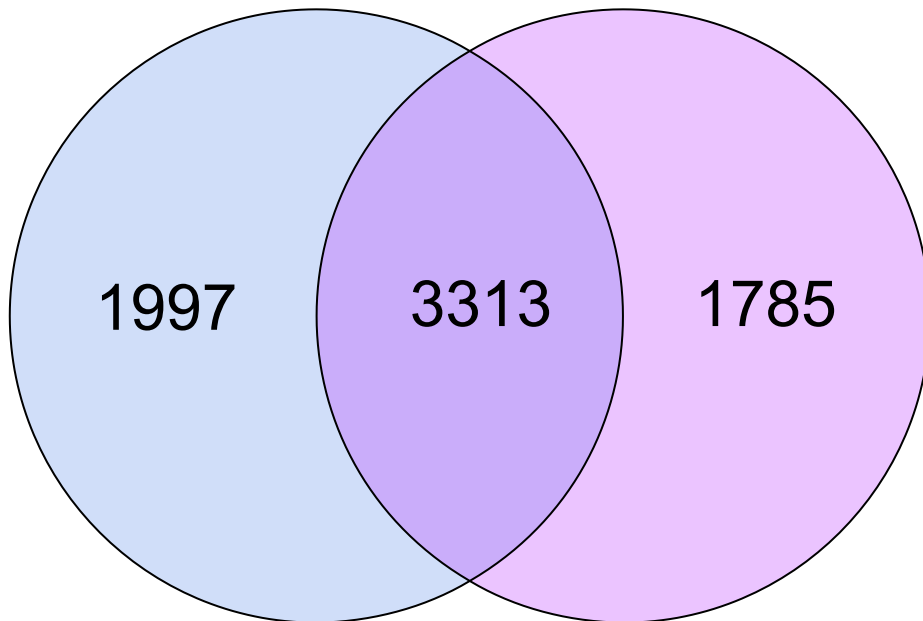

Supplement: Supplementary Figure S16 — Comparison of the Asian CNGB030001 benchmark and the GIAB HG002 benchmark within the overlapping benchmark regions The overlapping benchmark regions spanned 1.33 Gb on the genome. Both unique and overlapping SVs were identified. [file mmc16.pdf]

GO enrichment

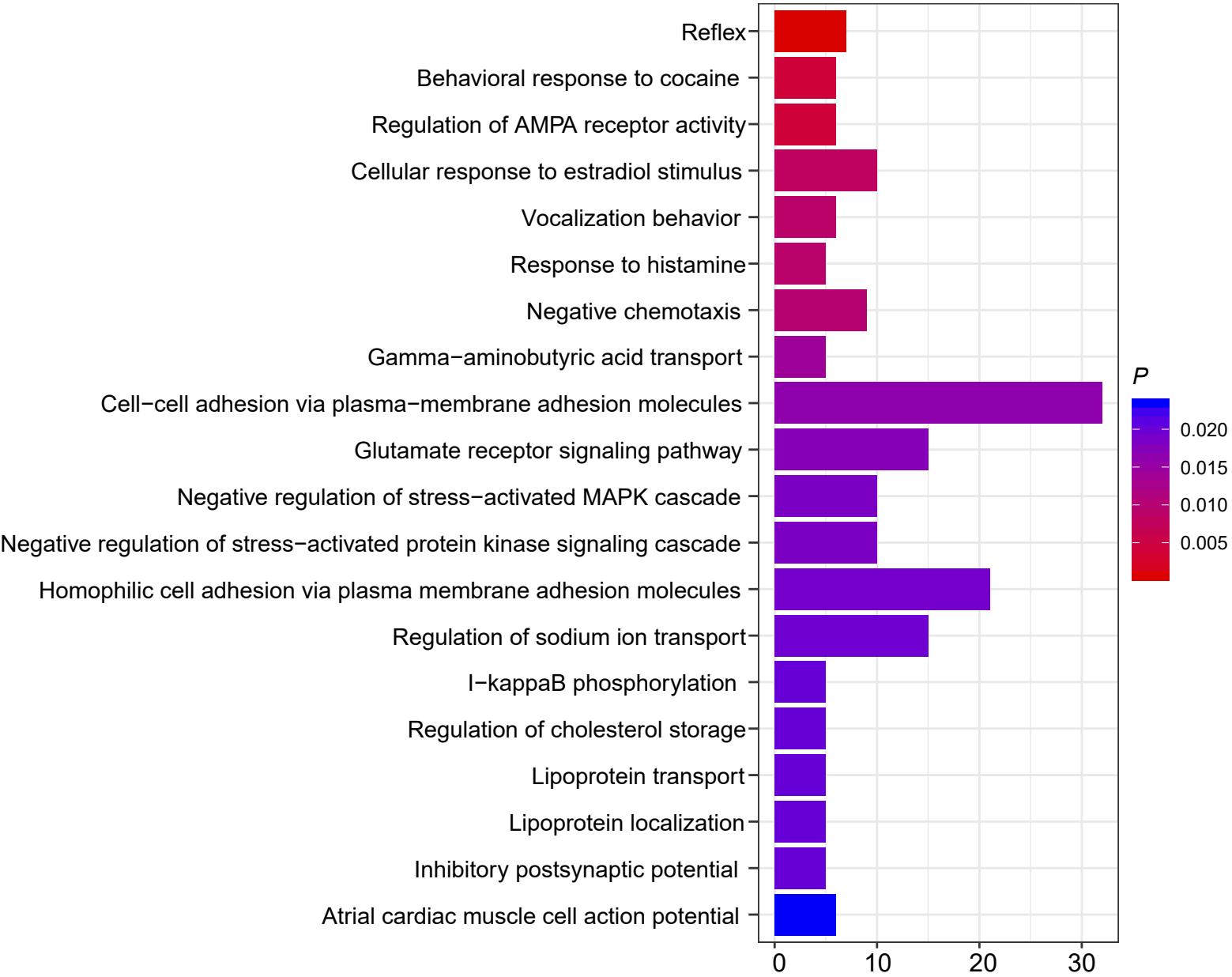

Supplement: Supplementary Figure S18 — GO enrichment of Asian-benchmark-specific SVs The Asian CNGB030001 benchmark was compared to the GIAB HG002 benchmark within their overlapping benchmark regions for identifying Asian-benchmark-specific SVs. [file mmc18.pdf]
