# Supplementary material for: Robust Benchmark Structural Variant Calls of An Asian Using State-of-the-art Long-read Sequencing Technologies
Source: Genomics Proteomics Bioinformatics. 2021 Mar 2;20(1):192–204. doi: 10.1016/j.gpb.2020.10.006 (PMC9510867; doi:10.1016/j.gpb.2020.10.006)
Supplement: Supplementary Figure S17 — Size distributions for common and Asian unique SVs The Asian CNGB030001 benchmark was compared to the GIAB HG002 benchmark within their overlapping benchmark regions. [file mmc17.pdf]

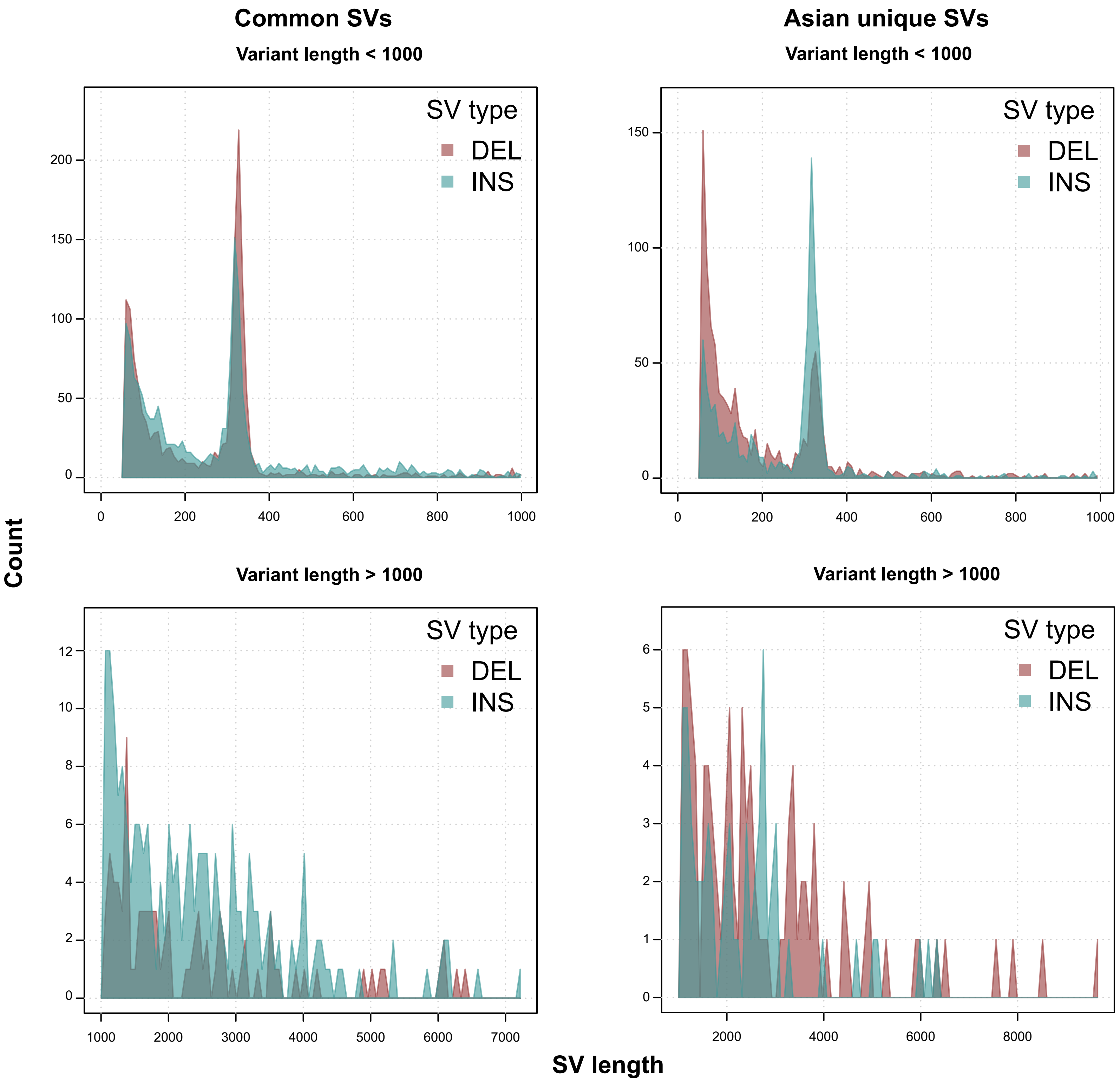

Common SVs
Asian unique SVs
Variant length < 1000
Variant length > 1000
SV type
DEL
INS
Count
SV length
