# Supplementary material for: Robust Benchmark Structural Variant Calls of An Asian Using State-of-the-art Long-read Sequencing Technologies
Source: Genomics Proteomics Bioinformatics. 2021 Mar 2;20(1):192–204. doi: 10.1016/j.gpb.2020.10.006 (PMC9510867; doi:10.1016/j.gpb.2020.10.006)
Supplement: Supplementary Table S1 — Counts of high-confidence SVs on each chromosome [file mmc19.docx]

**Table S1 Counts of high-confidence SVs on each chromosome**

| **Chromosome** | **Deletion count** | **Insertion count** | **Total count** |
| --- | --- | --- | --- |
| 1 | 318 | 356 | 674 |
| 2 | 342 | 353 | 695 |
| 3 | 234 | 247 | 481 |
| 4 | 333 | 263 | 596 |
| 5 | 230 | 198 | 428 |
| 6 | 297 | 307 | 604 |
| 7 | 281 | 264 | 545 |
| 8 | 244 | 241 | 485 |
| 9 | 169 | 204 | 373 |
| 10 | 257 | 229 | 486 |
| 11 | 178 | 230 | 408 |
| 12 | 207 | 231 | 438 |
| 13 | 169 | 198 | 367 |
| 14 | 120 | 121 | 241 |
| 15 | 99 | 129 | 228 |
| 16 | 129 | 130 | 259 |
| 17 | 148 | 151 | 299 |
| 18 | 130 | 119 | 249 |
| 19 | 148 | 150 | 298 |
| 20 | 121 | 140 | 261 |
| 21 | 83 | 101 | 184 |
| 22 | 75 | 87 | 162 |
| X | 75 | 91 | 166 |
| Y | 5 | 6 | 11 |
| Total | 4387 | 4540 | 8938 |
