# Supplementary material for: Robust Benchmark Structural Variant Calls of An Asian Using State-of-the-art Long-read Sequencing Technologies
Source: Genomics Proteomics Bioinformatics. 2021 Mar 2;20(1):192–204. doi: 10.1016/j.gpb.2020.10.006 (PMC9510867; doi:10.1016/j.gpb.2020.10.006)
Supplement: Supplementary Table S2 — Trio binning of CCS reads [file mmc20.docx]

**Table S2 Trio binning of CCS reads**

| ***k*-mer** | **Parental CCS reads** | **Maternal CCS reads** | **Unknown** | **hapA/hapB** | **Assigned percentage (%)** |
| --- | --- | --- | --- | --- | --- |
| 21 | 1,871,748 | 1,925,096 | 1,774,456 | 1.03 | 68.15 |
| 41 | 1,809,763 | 2,029,544 | 1,731,993 | 1.12 | 68.91 |
| 51 | 1,876,119 | 2,186,313 | 1,508,868 | 1.17 | 72.92 |
| 61 | 1,936,408 | 2,440,087 | 1,194,805 | 1.26 | 78.55 |
| 81 | 830,662 | 1,851,591 | 2,889,047 | 2.23 | 48.14 |
| Join | 1,941,539 | 2,374,195 | 1,255,566 | 1.22 | 77.46 |

*Note*: the subject’s PacBio CCS reads were partitioned into paternal- and maternal- inherited reads using the trio-binning strategy by integrating five different k-mers.
