# Supplementary material for: Robust Benchmark Structural Variant Calls of An Asian Using State-of-the-art Long-read Sequencing Technologies
Source: Genomics Proteomics Bioinformatics. 2021 Mar 2;20(1):192–204. doi: 10.1016/j.gpb.2020.10.006 (PMC9510867; doi:10.1016/j.gpb.2020.10.006)
Supplement: Supplementary Table S3 — Comparison between SVs from NGS platforms and benchmark SVs [file mmc21.docx]

**Table S3 Comparison between SVs from NGS platforms and benchmark SVs**

|  | **Insertion** | | | | **Deletion** | | | |
| --- | --- | --- | --- | --- | --- | --- | --- | --- |
|  | **MGISEQ**  **2000** | **%** | **NovaSeq**  **6000** | **%** | **MGISEQ**  **2000** | **%** | **NovaSeq**  **6000** | **%** |
| Manta | 1397 | 39.51% | 1000 | 28.28% | 1901 | 56.81% | 2100 | 62.76% |
| GRIDSS | 236 | 6.67% | 505 | 14.28% | 1372 | 41.00% | 1672 | 49.97% |
| LUMPY | 0 | 0.00% | 0 | 0.00% | 1282 | 38.31% | 1035 | 30.93% |
| BreakDancer | 39 | 1.10% | 5 | 0.14% | 1386 | 41.42% | 1277 | 38.16% |
| SV benchmark | 3536 |  |  |  | 3346 |  |  |  |

*Note*: in comparison to SV benchmark, overlapping SVs identified by different callers from MGISEQ2000 and NovaSeq 6000 were summarized. % refers to the corresponding overlapping rates.
